# Illumina error profiles: resolving fine-scale variation in metagenomic sequencing data

**DOI:** 10.1186/s12859-016-0976-y

**Published:** 2016-03-11

**Authors:** Melanie Schirmer, Rosalinda D’Amore, Umer Z. Ijaz, Neil Hall, Christopher Quince

**Affiliations:** The Broad Institute of MIT and Harvard, 415 Main Street, Cambridge, MA 02142 USA; Harvard T.H. Chan School of Public Health, 655 Huntington Ave, Boston, MA 02115 USA; University of Liverpool, Centre for Genomic Research, Crown Street, Liverpool, L69 7ZB UK; University of Glasgow, School of Engineering, Oakfield Avenue, Glasgow, G12 8LT UK; University of Warwick, Warwick Medical School, Coventry, CV4 7AL UK

**Keywords:** Sequencing errors, Sequencing-by-synthesis, Illumina, Metagenomics, Error profiles, Transposome bias

## Abstract

**Background:**

Illumina’s sequencing platforms are currently the most utilised sequencing systems worldwide. The technology has rapidly evolved over recent years and provides high throughput at low costs with increasing read-lengths and true paired-end reads. However, data from any sequencing technology contains noise and our understanding of the peculiarities and sequencing errors encountered in Illumina data has lagged behind this rapid development.

**Results:**

We conducted a systematic investigation of errors and biases in Illumina data based on the largest collection of *in vitro* metagenomic data sets to date. We evaluated the Genome Analyzer II, HiSeq and MiSeq and tested state-of-the-art low input library preparation methods. Analysing *in vitro* metagenomic sequencing data allowed us to determine biases directly associated with the actual sequencing process. The position- and nucleotide-specific analysis revealed a substantial bias related to motifs (3mers preceding errors) ending in “GG”. On average the top three motifs were linked to 16 % of all substitution errors. Furthermore, a preferential incorporation of ddGTPs was recorded. We hypothesise that all of these biases are related to the engineered polymerase and ddNTPs which are intrinsic to any sequencing-by-synthesis method. We show that quality-score-based error removal strategies can on average remove 69 % of the substitution errors - however, the motif-bias remains.

**Conclusion:**

Single-nucleotide polymorphism changes in bacterial genomes can cause significant changes in phenotype, including antibiotic resistance and virulence, detecting them within metagenomes is therefore vital. Current error removal techniques are not designed to target the peculiarities encountered in Illumina sequencing data and other sequencing-by-synthesis methods, causing biases to persist and potentially affect any conclusions drawn from the data. In order to develop effective diagnostic and therapeutic approaches we need to be able to identify systematic sequencing errors and distinguish these errors from true genetic variation.

**Electronic supplementary material:**

The online version of this article (doi:10.1186/s12859-016-0976-y) contains supplementary material, which is available to authorized users.

## Background

Illumina currently represents the dominant technology in the sequencing market. Therefore, a better knowledge of systematic errors in Illumina sequencing data is urgently required to derive accurate and meaningful results. Here, we tested and compared the most established Illumina platforms: the HiSeq 2000, the MiSeq and the Genome Analyzer II. The Genome Analyzer (GA) was the first Solexa/Illumina sequencing platform and launched in 2006. Although popular for many years, it has now been outperformed by newer sequencers that offer longer reads and higher throughput. We included the platform to determine to what extent biases in the GA persist in the newer Illumina technologies. The HiSeq is currently one of the most popular platforms. The newest version (HiSeq 2500) can produce up to 2 billion paired-end reads of 2 ×125 bp within ≤6 days (sequencing only) in high output mode. In rapid run mode the platform can achieve up to 300 million paired-end reads of 2 ×250 bp in ≤60 h (cluster generation + sequencing). Illumina’s benchtop sequencer, the MiSeq, can produce the longest reads with up to 2 ×300 bp. Cluster generation, sequencing and base calling takes approximately 55 h and results in up to 25 million paired-end reads.

Here, we extend our work on error profiles in amplicon data sets in connection with the MiSeq platform [1] to metagenomic data sets and included additional Illumina platforms as well as more low-input library preparation methods. Amplicon sequencing is an important tool to study microbial diversity and to identify the bacteria present in samples, however, 16S rRNA sequencing cannot reveal the functional capacities of the organisms. Metagenomics reveals information about the complete genomes of the organisms and offers insight into their functional abilities resulting in a much broader picture of the community. Further, any amplification step prior to library construction is optional and not necessarily required. In contrast, amplicon sequencing always requires several cycles of polymerase chain reaction (PCR) prior to the library preparation process representing an additional source of errors and potential biases. Our previous study identified library preparation and forward and reverse primer combination as the driving factors leading to distinct error patterns. For metagenomics, the DNA is extracted and directly prepared for sequencing omitting the initial PCR amplification step. Note however, that the library preparation method involves a limited cycle PCR amplification step for the tagmentation of the fragments.

Several library preparation methods are available nowadays and the different experimental techniques potentially introduce different biases into the sequencing data. The standard Illumina method for preparing sequencing libraries starts with the fragmentation of the template DNA by either sonication, nebulisation or shearing. This is followed by DNA repair and end polishing, plus ligation of platform specific adaptors. These adapters comprise flow cell adapters, that allow the fragments to bind to the flow cell surface, sequencing primers, required for the synthesis of the template during the sequencing, and optional indices for multiplexing. Illumina’s standard TruSeq sample preparation kit supports this workflow and is available with 48 indices that can be used for single or dual indexing. For the TruSeq method 1 *μ*g of input DNA is recommended [2]. However, most of the input material is lost during the library preparation and the method is time-consuming and labor intensive. Recently, a new technology was developed that combines these steps into one reaction. The Nextera transposome technology allows simultaneous fragmentation and tagmentation by using an adapted *in vitro* transposition. This method requires less input DNA and offers shorter preparation times [3]. The transposome consist of the transposase and a transposon complex with engineered transposon ends. The transposase catalyses the insertion of excised transposons into the template DNA resulting in random double stranded breaks. During this process the 3’ end of the transposon strands, including a unique adapter sequence, gets attached to the 5’ end of the target DNA. After the template DNA is labeled at the 5’ end, a complementary tag is added to the 3’ end using a polymerase extension. Platform specific sequencing adapters can additionally be added, and the sample can be enriched and bar-coded with the standard Illumina indices using limited-cycle PCR. Libraries can be prepared in 90 min and are optimised for 50 ng of input DNA. Further, the low input NexteraXT kit enables libraries prepared with only 1 ng of input DNA.

Parkinson et al. introduced another low input library preparation method promising libraries from picogram quantities [4] by using a modified transposome-mediated fragmentation technique. Their results indicate, that a similar coverage can be achieved with 20 pg compared to the coverage obtained from a standard library prepared with 1 *μ*g of DNA.

Previous studies have shown that Illumina errors are not random and that biases are likely to be related to sequence context. A general increase of errors towards the end of the reads has been observed as well as a strand bias [5–9]. However, no coherent patterns were identified across these studies. Dohm et al. and Nakamura et al. only analysed GAII data sets. The former was an early study investigating reads of 27–36 bp. They found that errors are frequently preceded by G [5]. Nakamura et al. reported sequence-specific errors related to inverted repeats and GGC sequences [6]. Furthermore, Minoche et al. observed positions with elevated error rates investigating five data sets sequenced on the GA and HiSeq and an overrepresentation of G’s in close vicinity to the errors. However, they report that there was no universal short motif that co-occurred with elevated error rates in their study. They also observed elevated rates of insertions and deletions in homopolymeric regions [7]. The most frequent errors observed by Meacham et al. was GGG to GGT [8]. A more recent study by Allhoff et al. was based on the analysis of four data sets and the biases they identified were platform dependent [9].

We conducted a comprehensive and systematic study of errors in Illumina sequencing data. Multiple Illumina platforms were investigated, including the GAII, HiSeq and MiSeq benchtop sequencer. We used a diverse mock community and various parameters for the library preparation including new transposome-based methods requiring only nanograms of DNA. Low input library preparation methods present a great advance for DNA sequencing as large quantities of input material are not always available. These methods make sequencing accessible to a broader range of research areas, including clinical and environmental studies as well as forensics. We analysed the errors and biases associated with these methods to test their capabilities and compare them to the standard library preparation method. We show that there are coherent patterns for all investigated Illumina sequencing platforms and that these systematic errors persist in newer sequencing platforms. Previously these errors have been ascribed to secondary structure and folding effects due to inverted repeats. Instead, we hypothesise that these errors are due to the engineered ddNTPs and polymerase which are the two key elements for any sequencing-by-synthesis method.

## Results

This section starts with a detailed discussion of the nucleotide- and position-specific error profiles for one HiSeq data set (*DS70*) and a comparison of the quality scores associated with the different types of errors. The same detailed analysis was conducted for all 23 MiSeq, 16 HiSeq and 13 GAII data sets (see Additional file 1: Table S1 for details). For the overall comparison of all 52 data sets, we also investigate the error rates with regards to the original nucleotide and substituting nucleotide. This is followed by a comparison of the motifs identified for each of the data sets. Furthermore, we examine the accuracy of the quality scores and their ability to predict different types of errors. This section concludes with an outline of the capacities of different error removal approaches across platforms and library preparation methods.

### Detailed error and quality profiles for data set *DS70*

Here, we present the detailed error profiles for *DS70*, one of the HiSeq data sets, where the library for the balanced mock community was prepared with the Nextera kit using 50 ng of input DNA (paired-end run, 2 ×100 bp). The substitution profiles of the metagenomic data set can be found in the Additional file 1: (Figure S1). The graphs highlight the tendency of substitutions to cluster together.

#### Error profiles

The overall error rates of this data set were very low: a rate of 0.0021 (errors per base) was recorded for the R1 reads and 0.0042 (errors per base) for the R2 reads. However, the errors are not evenly distributed across positions and nucleotides, creating a notable bias. The nucleotides A and C showed the lowest error rates with 0.0004 (errors per base) in the R1 reads and 0.0008 (errors per base) in the R2 reads for both nucleotides. G showed a slightly higher average error rate of 0.0005 (errors per base) in the R1 reads and 0.0010 (errors per base) in the R2 reads. T exhibited the highest average error rate with 0.0008 and 0.0015 (errors per base), respectively for R1 and R2. Further, much higher error rates were observed at individual positions. For example, at read position 35 in the R2 reads substitutions of T were observed at a rate of 0.0174 (errors per base). Overall, error rates increased towards the end of the read and errors are twice as likely to occur in R2 reads. In addition, we observed a clear bias in terms of the substituting nucleotide (see Additional file 1: Figure S1). G seems to be preferentially incorporated if an A, C or T is sequenced and if G is sequenced a T is falsely incorporated for the majority of substitutions.

Indel errors occurred at a much lower rate compared to substitutions: Rates of 2.8·10^−6^ (errors per base) for R1 insertions and 5.1·10^−6^ (errors per base) for R1 deletions were observed. For R2 we observed rates of 3.5·10^−6^ and 4.9·10^−6^ (errors per base), respectively for insertions and deletions. Indel errors were more evenly distributed across the length of the read, with a minor increase for the last 10bp, and seemed mostly independent of the read cycle. Deletions of all four nucleotides were observed at comparable rates and, similarly, insertion rates were comparable across all nucleotides with the exception of G insertions, where marginally higher rates were recorded. The insertion and deletion profiles as well as the distribution of unknown nucleotides can be found in Additional file 1: Figure S2.

Next, we analysed the quality scores for the different error types (see Additional file 1: Figure S3). Overall, the data sets displayed very high quality scores with an average of 37 and 35 for R1 and R2, respectively. A large part of the substitution errors were well characterised: 69 % of the R1 substitutions and 86 % of the R2 substitutions showed quality scores below 20. For insertions and deletions, on the other hand, the quality scores were meaningless as the majority of indel errors were assigned a very high quality score. Only 19 % of the R1 and 35 % of the R2 indel errors showed quality scores below 20.

### Overall comparison of error and quality profiles

In the following we compare the error rates as well as biases with regards to the substituting nucleotide and nucleotide distribution across all data sets. Furthermore, we examine the motifs (3mers preceding errors) associated with substitution and indel errors and examine the overall reliability of quality scores.

#### Substitution rates

**Fig. 1:**
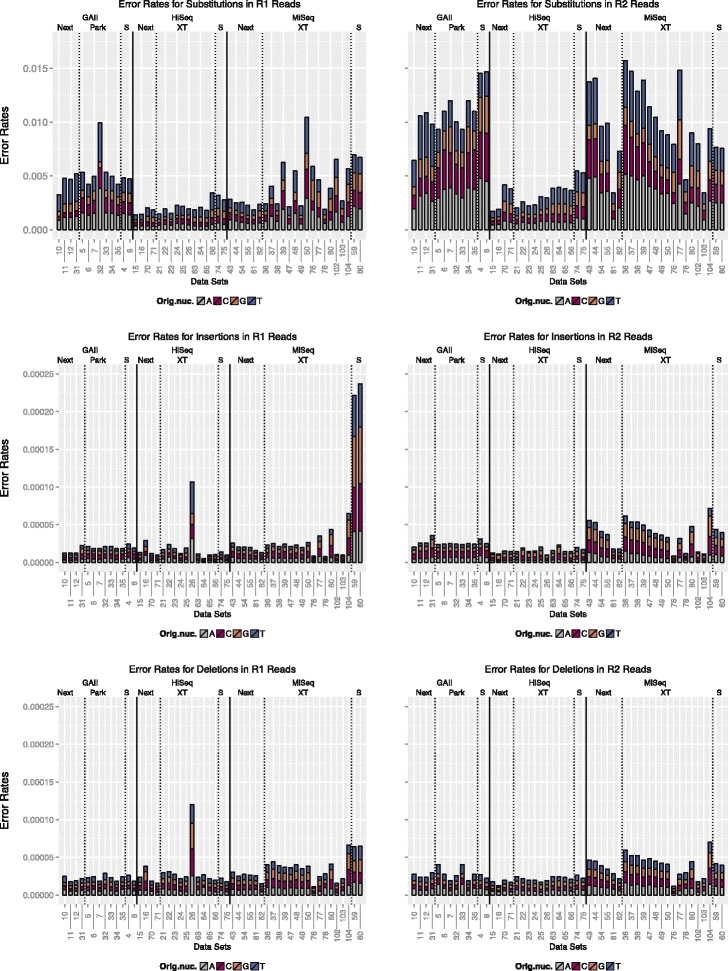
Comparison of error rates. The upper graphs indicate the proportion of substitutions of A, C, G and T for each data set, respectively. The two graphs in the middle show the proportion of inserted A, C, G and T nucleotides and the lower graphs show the proportion of deletions associated with each of the four nucleotides. Data sets are grouped by sequencing platform (*solid* lines) and library preparation method (*dashed* lines). Error rates are measured as errors per base

The overall error rates for all data sets are displayed in Fig. 1. The upper two graphs compare the substitution rates between platforms and library preparation method and show the differences between the R1 and R2 reads. The Genome Analyzer II (GAII) displayed the highest error rates with average substitution rates of 0.0051 (errors per base) for R1 reads and 0.0109 (errors per base) for R2 reads. The HiSeq data sets showed the lowest substitution rates of all three platforms with average rates of 0.0026 (errors per base) for R1 and 0.0040 (errors per base) for R2. The average error rates for the MiSeq, Illumina’s benchtop sequencer, were lower than the GAII but higher than the HiSeq error rates. Note however, that the MiSeq provides substantially longer reads than both HiSeq and GAII. We recorded average substitution rates in the MiSeq data sets of 0.0041 and 0.0099 (errors per base) for R1 and R2, respectively. The bar plots show the proportion of errors associated with the four different types of original nucleotides. For both the GAII and HiSeq, the highest substitution rates were observed for T and rates roughly doubled for the R2 reads. Additionally, the rates confirm that improvements for the HiSeq not only resulted in lower rates, but also in more similar substitution rates for A, C and G, however, the bias for T remains. For the MiSeq, R1 error rates were comparable for all for nucleotides. For R2 substitutions, higher rates were observed for A and T. (For further details see Table 1). Overall, the largest fluctuation in substitution error rates were recorded for the MiSeq.

**Table 1:** Average substitution rates for GAII, HiSeq and MiSeq. Substitution rates (∈ [0,1], errors per base) are split according to the original nucleotide

Platform	R1/R2	A	C	G	T
GAII	R1	0.0015	0.0010	0.0008	0.0018
GAII	R2	0.0035	0.0029	0.0019	0.0026
HiSeq	R1	0.0004	0.0004	0.0004	0.0008
HiSeq	R2	0.0007	0.0007	0.0007	0.0012
MiSeq	R1	0.0012	0.0009	0.0009	0.0012
MiSeq	R2	0.0033	0.0021	0.0015	0.0031

#### Indel rates

Insertion and deletion rates (displayed in Fig. 1) were generally very low. A sharp increase in insertions was observed for HiSeq data set *DS26* (Nextera XT, single organism, 2 ×101 bp paired-end reads) as well as the MiSeq data sets that were prepared with the standard library preparation method (see Additional file 1: Table S1 for details). For this HiSeq data set, two tight peaks in the position-specific insertion rates were recorded and for the two MiSeq data sets insertions accumulated over ≈25 bp in the centre of the reads.

#### Substituting nucleotide

In addition to recording the substituted nucleotides, we also analysed the substituting nucleotides that were falsely incorporated. Additional file 1: Figure S4 and S5 show the results for the R1 and R2 reads, respectively. For the GAII and HiSeq, C was rarely the substituting nucleotide in the R1 reads. A bias towards G was recorded for both R1 and R2 for all HiSeq data sets. One GAII data set (*DS32*) showed a high rate of G in the R1 reads. The nucleotides mainly affected by substitutions in this data set were T and A. For the MiSeq data sets a bias towards preferential incorporation of G and T was identified.

#### Motifs

**Fig. 2:**
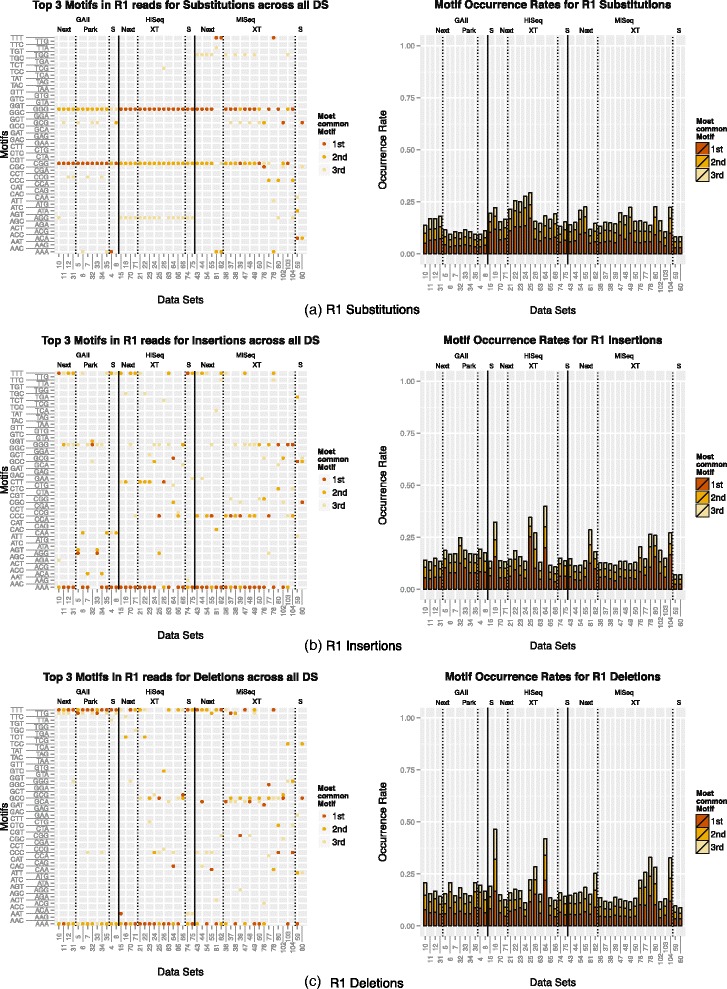
Motifs and occurrence rates: The top three motifs (3mers preceding errors) for R1 (**a**) substitutions, (**b**) insertions and (**c**) deletions are displayed on the *left*. The rates associated with each motif are displayed on the *right*. Data sets are grouped by sequencing platform and library preparation method

The motifs (3mers preceding errors) for all data sets were recorded. The results for the R1 reads are displayed in Fig. 2. For the R2 reads the analogous figure can be found in the Additional file 1: (Figure S6). We start by examining the motif-based nature of substitution errors for all three platforms. A coherent pattern for the substitution motifs was detected for all three platforms. The two most common motifs for both R1 and R2 reads for the GAII were “CGG” and “GGG”. On average, the first motif accounted for 4.7 % and 4.4 % of all substitutions in R1 and R2 reads, respectively, and the second motif accounted for 4.1 % in R1 and 3.9 % in R2 reads. For the HiSeq data sets the same two motifs were identified. Here, “GGG” was the first motif and “CGG” the second most common motif for every data set. The bias is more pronounced with on average 9.5 % and 10.0 % of all R1 and R2 substitutions, associated with the first motif. For some data sets more than 17 % of all R1 substitutions were associated with “GGG”. For the second motif on average 5.8 % of the R1 and 6.7 % of the R2 substitutions were preceded by this motif. The top three motifs accounted on average for 13.8 % and 14.8 % in the R1 and R2 reads, respectively. For the MiSeq data sets a strong bias associated with “GGG” and “CGG” was recorded, though more variation among the top motifs was observed. The top three motifs accounted on average for a total of 15.9 % of the R1 and 16.2 % of the R2 substitutions. We summarised the most common motifs for all three platforms in Table 2. It is notable, that all first and second motifs for all three platforms end in “GG”.

**Table 2:** Overview of the most common motifs for GAII, HiSeq and MiSeq

Platform	R1/R2	1st motif	2nd motif	3rd motif
GAII	R1	CGG	GGG	GCG
GAII	R2	CGG	GGG	CCG
HiSeq	R1	GGG	CGG	AGG
HiSeq	R2	GGG	CGG	AGG
MiSeq	R1	GGG	CGG	TGG
MiSeq	R2	GGG	CGG	GCG

The motifs associated with insertions showed a higher degree of variability, however 65 % of the top three motifs in the R1 reads were homopolymers and 52 % in the R2 reads. Overall, “AAA” was among the top three motifs in 42 data sets, “GGG” was observed 23 times, “TTT” 22 times and “CCC” 15 times for the R1 reads. We observed a similar bias for the R2 reads with “AAA” among the top three motifs in 24 data sets, “GGG” in 30, “TTT” in 16 and “CCC” in 10 data sets. The top three motifs accounted on average for 17.2 %/18.8 %/15.8 % of the R1 insertions and 14.0 %/15.3 %/15.1 % of the R2 insertions for GAII/HiSeq/MiSeq. However, the top three motifs accounted for as much as 40.0 % of R1 insertions and 28.5 % of R2 insertions in some data sets.

The two most common motifs in connection with deletion errors were “AAA” and “TTT”. In 38 data sets “AAA” was either the first or second most common motif and in 28 data sets “TTT” was among the two most common motifs in the R1 reads. For the R2 reads, “TTT” and “AAA” were recorded as the two most common motifs in 28 data sets, respectively. The third motif showed more variation across data sets. The top three motifs accounted on average for 17.2 %/19.9 %/17.0 % of the R1 insertions and 19.0 %/17.1 %/15.6 % of the R2 insertions for GAII/HiSeq/MiSeq. The maximum rate for all three motifs was 46.4 % and 46.7 % for R1 and R2 deletions, respectively.

The motif bias was observed regardless of the sample that was sequenced. These included two diverse mock communities as well as single organisms. However, to ensure that the observed biases are not a result of an over-representation of particular 3mers, we also computed the top three motifs while taking their frequencies into account. Additional file 1: Figure S17 shows that indeed a nonuniform distribution of the 64 possible 3mers is observed in the reads, with some 3mers occurring more frequently than others. Additional file 1: Figure S18 summarises the three most common motifs associated with errors taking the motif frequency based on the reads into account (i.e. total number of errors connected to a particular motif divided by the number of occurrences of this motif). For the majority of the data sets the top three motifs associated with substitutions were “GGG”, “CGG” and “AGG”. For insertions and deletions a higher degree of variation was observed.

#### Quality scores

**Fig. 3:**
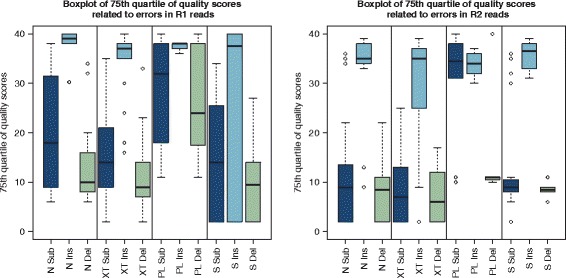
Quality scores: Overview of the 75th quartile of quality scores associated with errors across all data sets. The results for the R1 reads are displayed on the left and the results for the R2 reads are on the right. Data sets were grouped by library preparation method (N = Nextera, XT = NexteraXT, PL = Parkinson, S = Standard TruSeq) and substitution, insertion and deletion errors are displayed separately

Figure 3 displays the 75th quartile of the quality scores associated with errors for all data sets grouped by library preparation method and type of error. (The quality scores for the 50th quartile can be found in Additional file 1: Figure S7.) Insertions were generally poorly characterised by their quality scores for R1 and R2 reads for all library preparation methods and will not be identified by quality-score-based error correction methods. The quality scores for deletions were more reliable except for the R1 Parkinson data sets. For substitutions the 50th quartile for Nextera, NexteraXT and the standard TruSeq library preparation method was below 20 for all data sets (see Additional file 1: Figure S7). R2 substitution errors were generally better characterised than R1 substitution errors which is illustrated in Fig. 3. The 75th quartile of quality scores associated with substitutions shows higher values in connection with R1 reads compared to the R2 reads (except for the Parkinson data sets). For the Parkinson data sets 25 % of all substitutions as well as deletions on R1 reads were associated with high quality scores and therefore poorly represented.

#### Nucleotide distribution

**Fig. 4:**
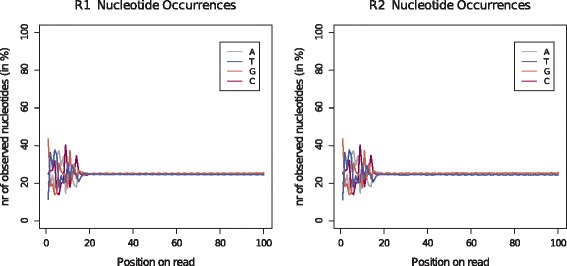
Nucleotide rates: Comparison of occurrence rates of the four nucleotides across the reads for data set *DS70*. The library for this data set was prepared with the Nextera kit and sequenced on the HiSeq

For all Nextera and NexteraXT libraries we observed uneven nucleotide distributions at the start of the reads. These library preparation methods rely on the transposome technology, where the transposase, is used to simultaneously fragment and tagment the template DNA. For most Nextera and NexteraXT data sets these fluctuations affected approximately the first 20 bp of the R1 and R2 reads. An example can be found in Fig. 4 and Additional file 1: Figure S8 displaying the results for data set *DS70*. The resemblance of the patterns for the R1 and R2 reads was particularly noticeable. For the Parkinson libraries, which use an adapted version of the Nextera technology, similar fluctuations were observed (see Additional file 1: Figure S9). Here, these fluctuations affected a larger part of the start of the read (30 bp). The observed fluctuations were also more pronounced. For further reference, an example for the standard TruSeq library preparation method is included in the Additional file 1: (Figure S10).

### Comparison of error rate removal techniques

**Fig. 5:**
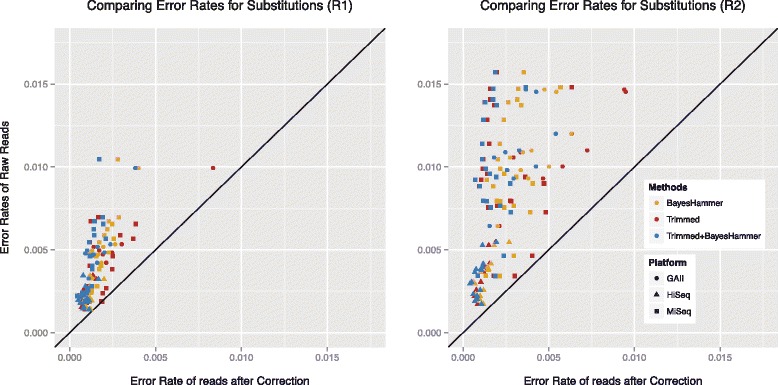
Comparison of error removal strategies for R1 and R2 reads: quality trimming with sickle (*red*), error correction with BayesHammer (*yellow*) and a combination of the two approaches (*blue*) was tested on all data sets. (Rates ∈ [0,1] are measured as errors per base.)

We tested two error removal strategies for the reads: quality trimming and error correction. For quality trimming the program sickle [10] (version 1.2000) with a minimum quality score of 20 and a minimum read length of 10 was used. For error correction we used the program BayesHammer which is part of the SPAdes assembler [11] (version 2.5.2). For the combination of both approaches the reads were first quality trimmed and then error corrected. Figure 5 displays the results for the R1 and R2 reads. Similar rates in error reduction were observed for R1 and R2. Averaged over all data sets, quality trimming reduced the R1 error rates by 54 % (GAII: 55 %, HiSeq: 50 %, MiSeq: 55 %) and R2 error rates by 64 % (GAII: 51 %, HiSeq: 66 %, MiSeq: 70 %). Error correction with BayesHammer reduced the R1 rates by 55 % (GAII: 61 %, HiSeq: 46 %, MiSeq: 58 %) and R2 rates by 65 % (GAII: 61 %, HiSeq: 61 %, MiSeq: 69 %). The best results, on average, across all platforms were achieved by combining the two approaches: R1 error rates decreased by 64 % (GAII: 67 %, HiSeq: 55 %, MiSeq: 67 %) and R2 rates decreased by 74 % (GAII: 69 %, HiSeq: 70 %, MiSeq: 80 %).

**Fig. 6:**
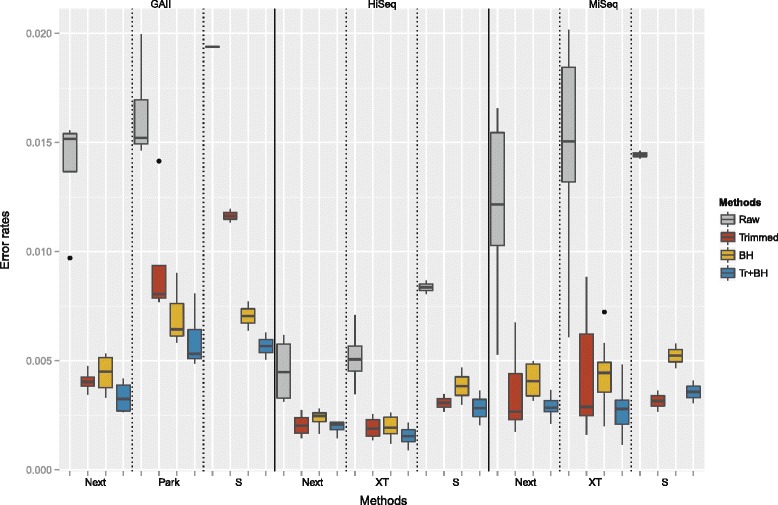
Comparison of error rates for different correction methods: Overall substitution error rates split by sequencing platform and library preparation method. The *grey* bars display the error rates of the raw reads. The *red* bars represent the error rates after quality trimming with sickle (minimum quality score: 20, minimum read length: 10) and the *yellow* bars represent the results after error correction with BayesHammer. The results of the combination of both methods are displayed by the *blue* bars. (Error rates ∈ [0,1] are measured as errors per base.)

Further, in Fig. 6 we compared the substitution error rates for the different library preparation methods for all platforms. The grey error bars represent the initial errors based on the raw reads. The highest error rates were encountered for the GAII, followed by slightly lower rates for the MiSeq and the lowest rates were observed for the HiSeq. For each platform, the data sets prepared with the Nextera library preparation method yielded the lowest error rates on average. The low input libraries, NexteraXT and Parkinson, resulted in slightly higher error rates, however, the highest error rates were observed for the standard library preparation for the GAII and HiSeq. For the MiSeq the highest raw error rates were associated with NexteraXT data sets. The results based on quality trimming are presented by the red bars. The greatest error reduction was observed for the standard and NexteraXT library MiSeq data sets. On average quality trimming worked better than error correction (represented by the yellow bars) except for the Parkinson and standard library GAII data sets. Generally, quality trimming followed by error correction (displayed in blue) yielded the best results and the error rate showed less variability. Only for the standard library MiSeq data sets did quality trimming without error correction result in slightly lower error rates. Overall, the HiSeq data sets exhibited the lowest error rates after trimming and error correction and the best results were achieved in connection with the Nextera and NexteraXT library preparation method. The MiSeq data sets showed comparable error rates after processing the reads and the best results were also achieved with the Nextera and NexteraXT library preparation method. Notably, the MiSeq read lengths are still considerably longer after error removal (see Table 3). The processed GAII data sets exhibited the highest error rates where the best results were also achieved in connection with the Nextera library preparation method.

**Table 3:** Average read length after trimming and error correction for the different platforms and library preparation methods (Nextera, NexteraXT (NXT), Parkinson, Standard)

Platform	GAII			HiSeq			MiSeq		
Library	Nextera	Parkinson	Standard	Nextera	NXT	Stand.	Nextera	NXT	Standard
Original	100	101	101	100/101	100/101	100	250	250	250
Quality trimming	89	92	92	97	96	94	209	205	175
BayesHammer	92	94	95	98	98	96	219	216	187
Both	90	93	94	97	96	94	210	206	176

#### Aligned reads

**Fig. 7:**
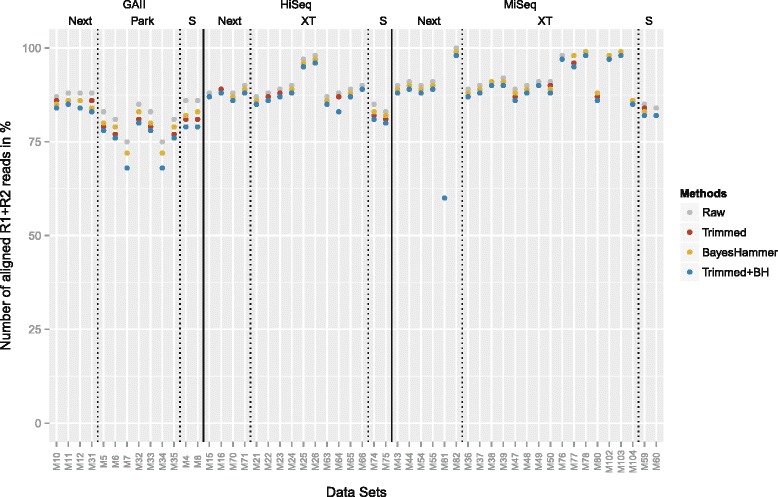
Comparison of aligned R1+R2 reads: Percentage of aligned reads for the raw data sets, after quality trimming, error correction and the combination of the two approaches (Next=Nextera, XT=NexteraXT, Park=Parkinson, S=Standard TruSeq)

All error rates and calculation are based on aligned reads. Figure 7 shows the percentage of aligned reads for all data sets and Table 4 shows the average rates for the raw reads across all sequencing platforms and library preparation methods. Overall, very good alignment rates were attained for all methods. The highest rates for each platform were obtained for the NexteraXT libraries sequenced on the HiSeq and MiSeq and the Nextera libraries sequenced on the GAII. The percentage of aligned reads slightly decreased after quality trimming and error correction, as reads may be shortened or discarded by the programs. After trimming and subsequent error correction 2.6–7.4 % less GAII reads aligned, 1.2–5.5 % less HiSeq reads and 0.2–2.9 % less MiSeq reads.

**Table 4:** Average percentage of aligned raw reads for the different platforms and library preparation methods (Nextera, NexteraXT, Parkinson, Standard)

GAII			HiSeq			MiSeq		
Nextera	Parkinson	Stand.	Nextera	NXT	Stand.	Nextera	NXT	Stand.
87.7 %	80.6 %	85.9 %	88.8 %	90.4 %	84.1 %	87.0 %	92.6 %	84.4 %

## Discussion

**Fig. 8:**
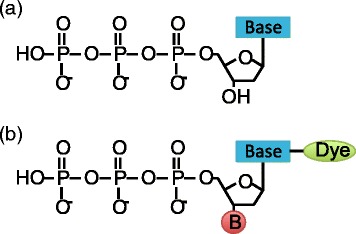
Chemical formula of dNTPs and ddNTPs: **a** Deoxynucleotides (dNTPs): natural nucleoside triphosphates that get incorporated during DNA polymerase. **b** Reversible dye-terminators: engineered nucleotides used for Illumina sequencing-by-synthesis

**Fig. 9:**
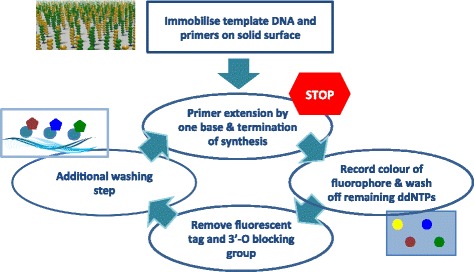
Overview of Illumina sequencing using reversible dye-terminating ddNTPs: The template DNA sequences including the primers are first immobilised on a solid surface. During each cycle the polymerase incorporates one reversible dye-terminator base (ddNTP). The synthesis is temporarily paused and the dye is excited with a laser to identify the incorporated nucleotide. All remaining ddNTPs are washed off and the fluorescent tag and 3’-O blocking group is removed. This is followed by another washing step before the DNA polymerase recommences

For sequencing-by-synthesis methods, such as Illumina, the DNA polymerase is a key element. The *E. coli* DNA polymerase I (Pol I) proteolytic (Klenow) fragment was the first polymerase used for Sanger sequencing and the only DNA polymerase available at the time. Fortunately, this polymerase permits the incorporation of chain termination dideoxynucleotides (ddNTPs) which inhibit the DNA synthesis and form another key element for this sequencing method. Unlike natural dNTPs, the ddNTPs lack the 3’-hydroxyl (3’-OH) group that is required for the phosphodiester bond formation between the incorporating nucleotide and primer terminus. Therefore, the DNA polymerase terminates after the incorporation of a ddNTP. Different fluorescent labels are covalently attached to each of the four ddNTPs enabling automated DNA sequencing and single tube reactions. Further advances included the replacement of the 3’-hydroxyl group with a larger, cleavable chemical group facilitating the reversible termination of the DNA synthesis and facilitating the current NGS sequencing-by-synthesis methods (see Fig. 8). An overview of the Illumina sequencing process can be found in Fig. 9.

Changing the 3’-OH group results in a modified moiety and makes it harder for the DNA polymerase to accept the engineered nucleotides. The original Klenow enzyme was not capable of efficiently incorporating these modified nucleotides, creating a need for a new enzyme. Sequencing information facilitated the discovery of multiple DNA polymerases from mesophilic/thermophilic viruses, bacteria and archaea and greatly advanced the search for a new enzyme suitable for sequencing-by-synthesis methods.

### Biases associated with ddGTPs

The *Thermus aquaticus* (*Taq*) DNA polymerase has been a commonly used enzyme for DNA sequencing as the *Taq* pol is readily purified, thermostable and can be further modified. The original enzyme incorporates ddNTPs much more slowly than dNTPs. A mutation (F667Y) greatly increased the efficiency of ddNTP incorporations. However, the *Taq* pol enzyme favours the incorporation of ddGTP over the other ddNTPs, due to interactions between the guanidinium side chain of the arginine residue 660 (Arg660) and O6/N7 atoms of the guanine base. A substitution in the Arg660 residue with a negatively charged aspartic acid, aims at remediating this bias [12]. However, this is no longer achieved if the larger reversible dye-terminators are used. Development of different 3’-O-blocking groups has been an active field of research. Illumina/Solexa developed the 3’-O-azidomehtyl 2’-deoxynucleoside triphosphates and a mutant of the archaeal 9°N DNA polymerase of the hyperthermophilic *Thermococcus sp. 9°N-7* is used during sequencing. Limited information is available on the exact mutations due to commercial considerations [13–15]. However, for all data sets we still observed a dominant bias towards the incorporation of ddGTPs.

Not only preferential incorporation but also ten times faster incorporation rates for ddGTPs have been reported for the *Taq* DNA polymerase [12]. Li et al. subsequently studied the crystal structure for the different ddNTPs. The ddGTP ternary structure differs from the other ddNTPs as it possesses an additional hydrogen bond between the side chain of the Arg-660 residue and the base of the ddGTP complex. A mutation of the ARG-660 can reduce the incorporation rate of ddGTP and resolved the problem for Sanger sequencing methods [13–15]. We hypothesise that a similar bias occurs in connection with the archaeal 9°N DNA polymerase resulting in higher error rates after multiple ddGTPs have been incorporated which results in a bias towards motifs ending in “GG”. On average, 16 % of all substitution errors can be associated with only three motifs.

A non-uniform distribution of the 64 possible motifs was observed which is likely due to the biological significance of 3mers. Codons consist of three nucleotides and correspond to a particular amino acid which form the building blocks of proteins. To confirm that the observed motif bias is not simply a result of an over-representation of particular motifs in the data, we also took the motif frequency into account. This provided further support for the motifs identified in connection with substitution errors which are the main type of errors for Illumina sequencing. For insertions and deletions the top three motifs associated with these errors showed a larger degree of variation after normalising for motif frequency. This suggest that although more insertions and deletions are connected to certain motifs, that this might be due to an over-representation of these motifs in the data rather than a systematic error occurring during the sequencing process.

While some of the observed errors might be due to clonal mutations, the error profiles were independent of the sequenced input material suggesting that clonal mutations do not account for the detected motif bias. We sequenced several single organisms as well as a diverse mock community with different abundance distributions. If the error profiles were driven by clonal mutations, this would be reflected in the error distributions as these would depend on the sequenced sample. However, the motif bias was observed independent of the sequenced input material.

### Increased error rates towards the end of the reads and accumulation of errors

The individual error profiles confirmed an increase of the error rates towards the end of the reads, which has been previously reported and is attributed to an accumulation of phasing and pre-phasing problems during the run. The chemical and structural properties associated with the ddNTPs seem to contribute to this effect. After cleavage of the linker group carrying the fluoresphore, extra chemical molecules on the normal purine and pyrimidine bases remain and result in a vestige. These vestiges can perturb the DNA polymerase and limited the possible read length as they impair the stability of the DNA and hinders the substrate recognition and primer extension. Chen et al. [15] described an accumulation of these vestiges in Illumina sequencing. Illumina has been able to achieve longer reads by adding reversible terminator nucleotides without the fluorophore to reduce the effect of vestiges, but their impact is still apparent as increased error rates towards the end of the reads. We hypothesise that these vestiges encourage the accumulation of errors.

### Transposome bias

Furthermore, a bias associated with the transposome-based library technologies (Nextera, NexteraXT and Parkinson) was recorded, confirming previous findings in the context of transposons [16]. The transposase used for this technology is based on a mutated Tn5 transposome [17]. Transposons are capable of inserting themselves into a target DNA sequence. The wildtype Tn5 enzyme has been described as inactive [18], however, the mutations resulted in an increased insertion rate making the enzyme suitable for library preparations. For the wildtype Tn5, hot spots for insertions have been reported. The enzyme contains 19bp target recognition sites that are present at the ends of the transposase (Tnp), a protein that is part of the transposon complex and responsible for the catalytic steps. The target recognition sites are required in order for the transposon to bind to the template DNA for the subsequent insertion and it has been hypothesised that specific contacts must be formed between Tnp and the target DNA [19]. Ason et al. [19] observed high frequencies of insertions into A/T rich regions (in particular TTATA) flanked by GC pairs. As the recognition sequence of Tnp contains the same subsequence (TTATA), they suggest that Tnp favours insertions into regions containing a portion of the recognition site. Our data suggests that the mutated Tn5 enzyme used in the Nextera technology, shows a similar bias accounting for the uneven distribution at the start of the reads. The length of the fluctuations concurs with the length of the recognition site and higher rates of A/T were observed in the first part of this region followed by elevated G/C rates. However, this bias was not associated with errors and therefore these fluctuation do not need to be removed by trimming the start of the reads. It needs to be determined though if this tendency results in a coverage bias of the sequenced genomes and/or the coverage of the genomes in the community.

The Nextera method has many apparent advantages: it requires less DNA input material and the template DNA is simultaneously fragmented and tagmented facilitating shorter preparation times. A limited-cycle PCR step is involved in the tagmentation step, therefore, higher error rates were expected for the Nextera data sets. However, for all three platforms the data sets prepared with the Nextera kit showed the lowest error rates (see Fig. 6).

## Conclusion

Metagenomics has become widely available facilitating studies of fine-scale variation. Single-nucleotide polymorphisms in the human genome can cause life-threatening diseases, pathogenic and non-pathogenic bacterial strains often differ by only a few nucleotides and a small number of mutations can have disastrous effects on the virulence of an infection. In order to develop effective diagnostic and therapeutic approaches we need to be able to accurately characterise and identify systematic sequencing errors and distinguish these errors from true genetic diversity.

We showed that quality scores can characterise the majority of substitution and deletion errors for Nextera, NexteraXT and the TruSeq library preparation method. However, quality scores are meaningless for insertions. Insertion and deletion rates are 1000 times lower than substitution rates and therefore less significant. For applications where low frequency variants are important, the motifs identified in connection with indel errors can be used as further indication for the reliability of observed SNPs. Quality trimming (sickle) combined with subsequent error correction (BayesHammer) provided the best results in terms of error removal. The number of aligned reads decreased on average by 3 % which is likely due to shortening and discarding of some reads during the different error removal strategies, however, error rates could be reduced by as much as 89 %. On average substitution error rates were reduced by 69 %.

Any experimental procedure has the potential to introduce biases and errors. Comparing the different library preparation methods, the best accuracy was observed for the Nextera and NexteraXT methods. This technology facilitates simultaneous fragmentation and tagmentation of the DNA sample, resulting in shorter preparation times. In addition less input DNA is required for these methods. In connection with the proposed error removal strategy, we were able to reduce the error rates of the longer MiSeq reads to a level comparable to the HiSeq reads. This accentuates the MiSeq benchtop sequencer and the Nextera library preparation method as an excellent option for sequencing applications.

Current methods are not designed to target systematic errors in Illumina sequencing. Additional file 1: Figures S11–S16 and Tables S2 and S3 show that a strong bias remains after error removal based on quality scores. In order to achieve any further error reduction novel methods that specifically target these idiosyncrasies are required and will facilitate more accurate and detailed analysis of fine-scale variation in Illumina sequencing data.

## Methods

### Library preparation methods

The standard Illumina indices were used for all libraries. For the standard and Parkinson libraries multiplexing was implemented with single indices. For Nextera and NexteraXT dual indexing was employed. DNA quantities of 250 ng and 500 ng were tested for the standard library. Most Nextera libraries were prepared with 50 ng of starting DNA. In addition, two libraries with 0.5 ng were tested. The starting amount for the NexteraXT libraries was 1 ng for all data sets. For the Parkinson libraries 0.5 or 0.05 ng were used. After fragmenting the DNA a range of 600–900 bp was selected for all data sets with the Pippin Prep.

### Samples

The samples for sequencing included a diverse mock community consisting of 49 bacterial and 10 archaeal genomes. For the first mock community even amounts of genomic DNA were combined (balanced mock), for the second community the genomic DNA was mixed according to a log-normal distribution (unbalanced mock). For further details see [1]. We also sequenced several single species samples including *Burkholderia xenovorans (LB400)*, *Desulfovibrio desulfuricans subsp. desulfuricans str. ATCC 27774*, *Enterococcus faecalis V583*, *Nanoarchaeum equitans Kin4-M*, *Rhodospirillum rubrum ATCC 11170*, *Thermus thermophilus HB8* and *Treponemavincentii I*.

### Platforms

We tested the Genome Analyzer II, the HiSeq and the MiSeq. The samples sequenced on the MiSeq included 14 mock community samples and nine single species samples. The samples were prepared with Nextera, NexteraXT or the standard library preparation method. With higher throughput the HiSeq and GAII are more commonly used for diverse data sets where a high coverage is required. Therefore, we mainly tested the mock communities on these platforms. On the GAII we sequenced 13 mock community samples. These data sets covered the standard, Nextera and Parkinson libraries with varying amounts of starting DNA. The HiSeq data sets include 14 mock community samples and two single species samples and were prepared with the Nextera, NexteraXT and standard library preparation method. The samples were distributed across five runs on two MiSeq sequencers, two HiSeq runs and three GAII runs. Additional file 1: Table S1 provides an overview of the different parameters for each test data set. For very large data sets the reads were subsampled to one million for the MiSeq data sets, four million for the HiSeq data sets and three million for GAII data sets for the subsequent analysis.

### Reference database

The mock community was part of a study by Shakya et al. [20] which provided the majority of the genome sequences for our reference database. However, four organisms exhibited poor coverage and were therefore resequenced: *Desulfovibrio desulfuricans desulfuricans ATCC 27774*, *Enterococcus faecalis V583*, *Nanoarchaeum equitans Kin4-M* and *Treponema vincentii I*. The respective reads were assembled with VelvetOptimiser [21] and SPAdes [11]. Contigs of at least 1000 bp were concatenated and included in the reference database.

### Algorithm for computing the error profiles

The reads were aligned with the latest Burrows-Wheeler Aligner (BWA) algorithm in paired-end mode: BWA-MEM (version 0.7.9a) [22]. We used the -M option to mark shorter split hits as secondary alignments. All secondary alignments and unmapped reads were discarded. Our previous study on amplicon error profiles showed that the R1 and R2 reads exhibited distinct error patterns [1]. The paired-end alignment strategy was used as it offers higher accuracy, but for the subsequent analysis the aligned reads were again separated into R1 and R2 reads based on the FLAG field of the Sequence Alignment/Map (SAM) files. The FLAG field also specifies if the read originated from the plus or minus strand which was taken into consideration. Next, the MD tag was generated with SAMtools (version 0.1.18 and 0.1.19) [23]. We then applied the same algorithm as described in [1] to compute the position- and nucleotide-specific error profiles and motifs. Briefly, mismatches, insertions and deletions were identified based on the Compact Idiosyncratic Gapped Alignment Report (CIGAR) string, which also specifies the substituting nucleotides as well as the type of inserted nucleotides. The original nucleotides that were replaced during the substitutions were inferred from the MDtag in addition to the type of deleted nucleotides. The program outputs 4xL matrices for each error type (where L is the read length) for the R1 and R2 reads, respectively, where each row specifies the nucleotide-specific error rates for a certain position on the read.

## Availability

The code is available on: https://bitbucket.org/ms_research/ep_metagenomic. The data sets supporting the results of this article are available in the European Nucleotide Archive repository. Study accession number: PRJEB12287.

## Additional file

Additional file 1 Data further supporting the results including additional figures (Figures S1-S18) and tables (Tables S1-S3). (PDF 2808 kb)
