# Supplementary material for: Illumina error profiles: resolving fine-scale variation in metagenomic sequencing data
Source: BMC Bioinformatics. 2016 Mar 11;17:125. doi: 10.1186/s12859-016-0976-y (PMC4787001; doi:10.1186/s12859-016-0976-y)
Supplement: Additional file 1 — Data further supporting the results including additional figures (Figures S1-S18) and tables (Tables S1-S3). (PDF 2808 kb) [file 12859_2016_976_MOESM1_ESM.pdf]

Supplementary material

Table S1 Overview of the experimental design for the metagenomic data sets (1). Library preparation methods: Nextera (N), NexteraXT (XT), Parkinson Low Input (P), Standard TruSeq (S); Templates: *Burkholderia xenovorans (LB400)* (BX), *Desulfovibrio desulfuricans subsp. desulfuricans str. ATCC 27774* (DSV), *Enterococcus faecalis V583* (EF), *Nanoarchaeum equitans Kin4-M* (NE), *Rhodospirillum rubrum ATCC 11170* (RHO), *Thermus thermophilus HB8* (TT), *Treponema vincentii I* (TV), balanced mock community (MB), unbalanced mock community (MUB);

Platform	Meta ID	Library	Run	Machine	Input ng	Template	Read length
**MiSeq**	36	XT	1	Miseq1	1	MB	2x250bp
	37	XT	1	Miseq1	1	MB	2x250bp
	38	XT	1	Miseq1	1	MUB	2x250bp
	39	XT	1	Miseq1	1	MUB	2x250bp
	43	N	1	Miseq1	50	MB	2x250bp
	44	N	1	Miseq1	50	MUB	2x250bp
	47	XT	2	Miseq2	1	MB	2x250bp
	48	XT	2	Miseq2	1	MB	2x250bp
	49	XT	2	Miseq2	1	MUB	2x250bp
	50	XT	2	Miseq2	1	MUB	2x250bp
	54	N	2	Miseq2	50	MB	2x250bp
	55	N	2	Miseq2	50	MUB	2x250bp
	59	S	3	Miseq2	250	MB	2x250bp
	60	S	3	Miseq2	250	MUB	2x250bp
	76	XT	4	Miseq2	1	BX	2x250bp
	77	XT	4	Miseq2	1	DSV	2x250bp
	78	XT	4	Miseq2	1	EF	2x250bp
	80	XT	4	Miseq2	1	TT	2x250bp
	81	N	4	Miseq2	50	NE	2x250bp
	82	N	4	Miseq2	50	TV	2x250bp
	102	XT	5	Miseq2	1	BX	2x250bp
	103	XT	5	Miseq2	1	RHO	2x250bp
	104	XT	5	Miseq2	1	TT	2x250bp
**GAII**	4	S	1	GAII1	500	MB	2x101bp
	5	P	2	GAII1	0.5	MB	2x101bp
	6	P	2	GAII1	0.05	MB	2x101bp
	7	P	2	GAII1	0.05	MB	2x101bp
	8	S	2	GAII1	500	MUB	2x101bp
	10	N	3	GAII1	0.5	MUB	2x100bp
	11	N	3	GAII1	50	MUB	2x100bp
	12	N	3	GAII1	50	MB	2x100bp
	31	N	3	GAII1	0.5	MB	2x100bp
	32	P	2	GAII1	0.5	MB	2x101bp
	33	P	2	GAII1	0.5	MB	2x101bp
	34	P	2	GAII1	0.05	MB	2x101bp
	35	P	2	GAII1	0.05	MB	2x101bp
**HiSeq**	15	N	1	Hiseq1	50	MB	2x100bp
	16	N	1	Hiseq1	50	MUB	2x101bp
	21	XT	1	Hiseq1	1	MB	2x101bp
	22	XT	1	Hiseq1	1	MB	2x101bp
	23	XT	1	Hiseq1	1	MUB	2x101bp
	24	XT	1	Hiseq1	1	MUB	2x101bp
	25	XT	1	Hiseq1	1	DSV	2x101bp
	26	XT	1	Hiseq1	1	RHO	2x101bp
	63	XT	2	Hiseq1	1	MB	2x100bp
	64	XT	2	Hiseq1	1	MB	2x100bp
	65	XT	2	Hiseq1	1	MUB	2x100bp
	66	XT	2	Hiseq1	1	MUB	2x100bp
	70	N	2	Hiseq1	50	MB	2x100bp
	71	N	2	Hiseq1	50	MUB	2x100bp
	74	S	2	Hiseq1	250	MB	2x100bp
	75	S	2	Hiseq1	250	MUB	2x100bp

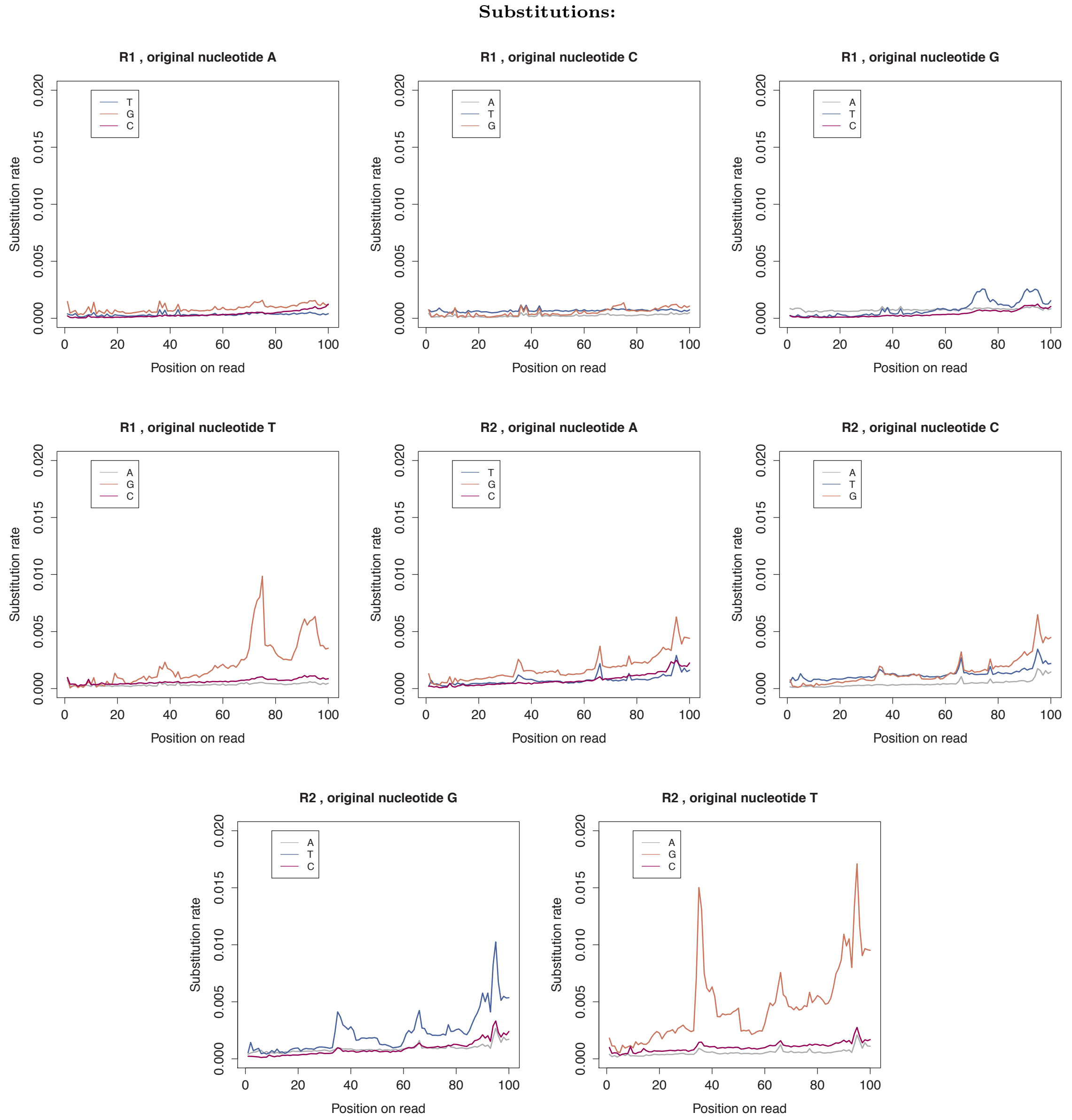

Figure S1 Nucleotide specific substitution error profiles for metagenomic data set *DS70*: Each graph shows the substitution rates for a specific original nucleotide and the colours indicate the substituting nucleotide. The first four graphs show the R1 profiles and the last four graphs show the R2 profiles. (Error rates $\in [0, 1]$ are measured as errors per base.)

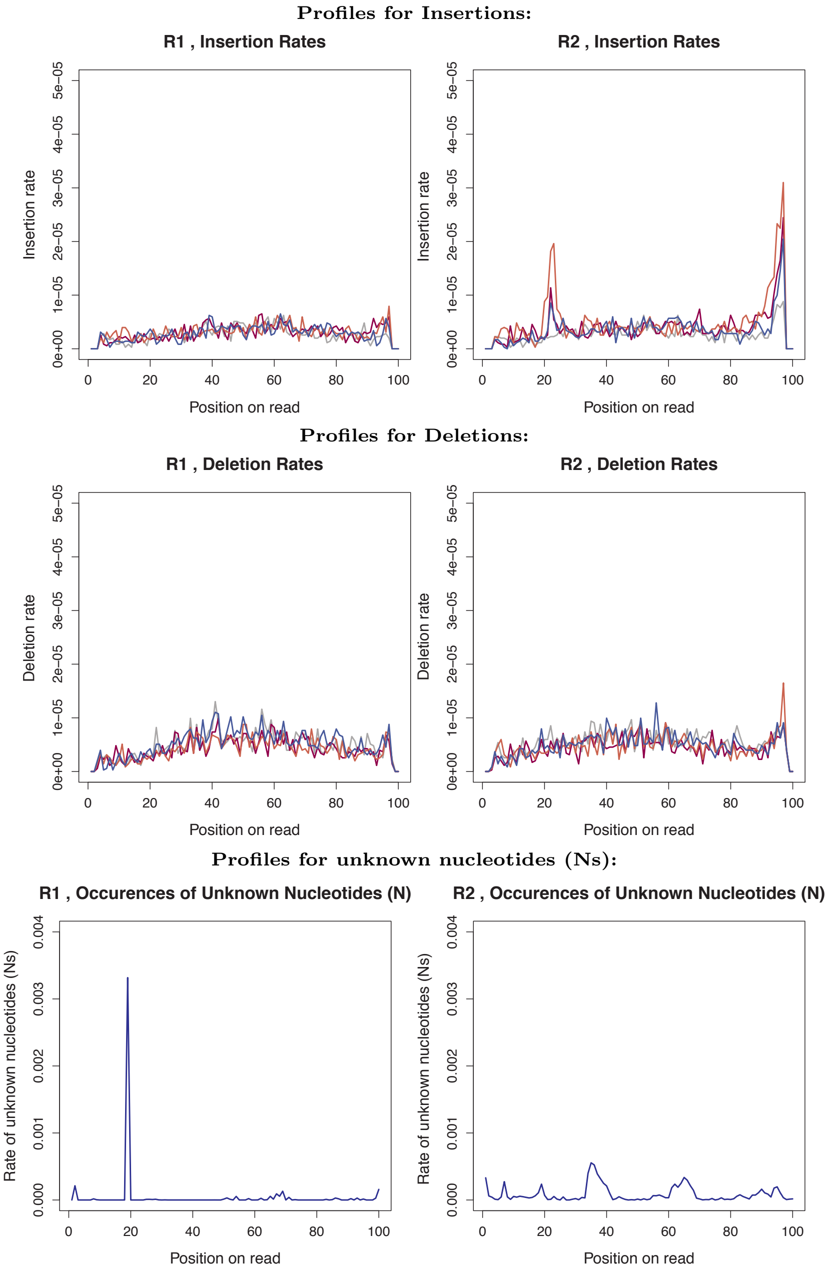

Figure S2 Error profiles for insertions, deletions and unknown nucleotides (Ns): The three graphs on the left show the R1 error profiles. For insertions, the colour identifies the inserted nucleotide and for deletions the colour refers to the type of nucleotide that was deleted. The three graphs on the right display the error profiles for the R2 reads, respectively. Rates are measured as nucleotides per base.

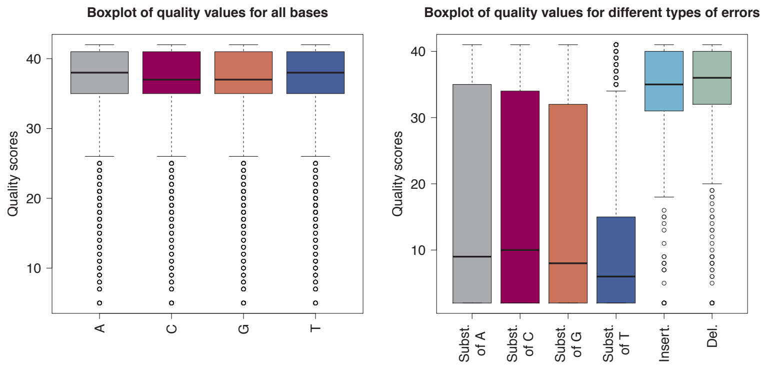

(a) R1 quality profiles

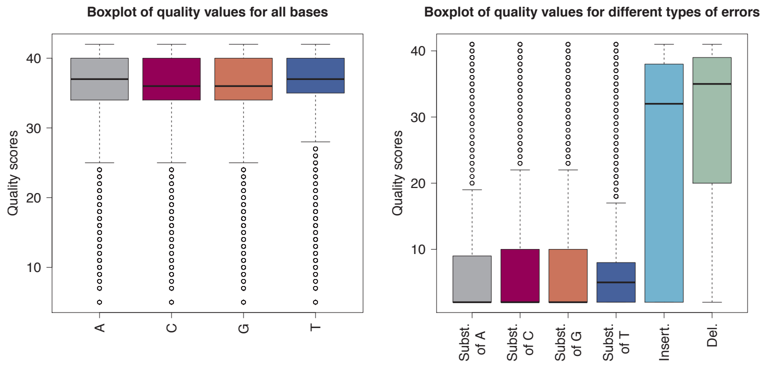

(b) R2 quality profiles

Figure S3 Quality profiles for R1 and R2 reads: The box plots in the first column display the distribution of quality scores for all bases. The second column shows the distribution of quality scores associated with errors.

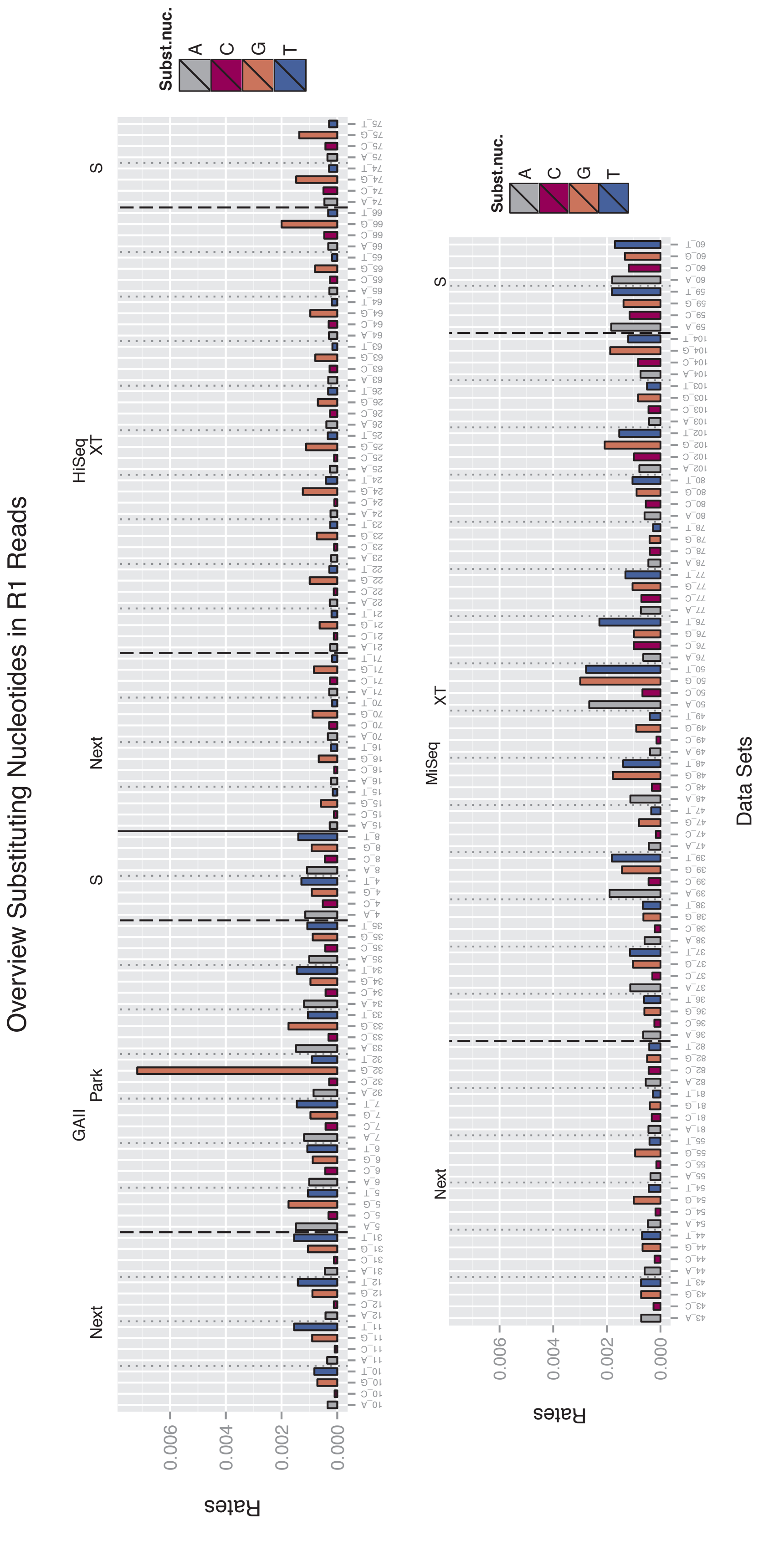

Figure S4 Comparison of substituting nucleotides in R1 reads: the upper plot shows the GAII and HiSeq, the lower plot shows the MiSeq data sets. (Rates $\in [0, 1]$ are measured as nucleotides per base.)

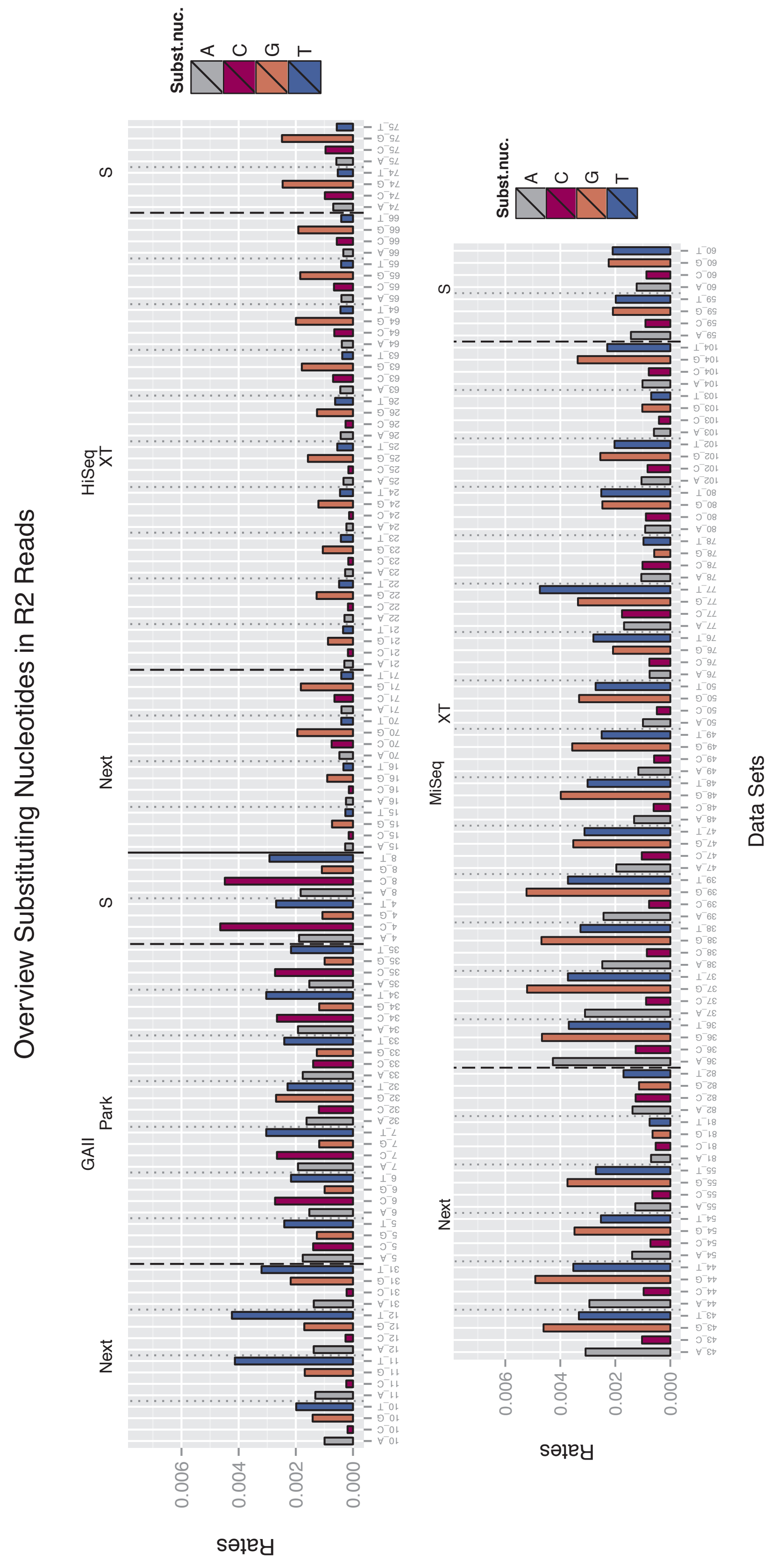

Figure S5 Comparison of substituting nucleotides in R2 reads: the upper plot shows the GAII and HiSeq, the lower plot shows the MiSeq data sets. (Rates $\in [0,1]$ are measured as nucleotides per base.)

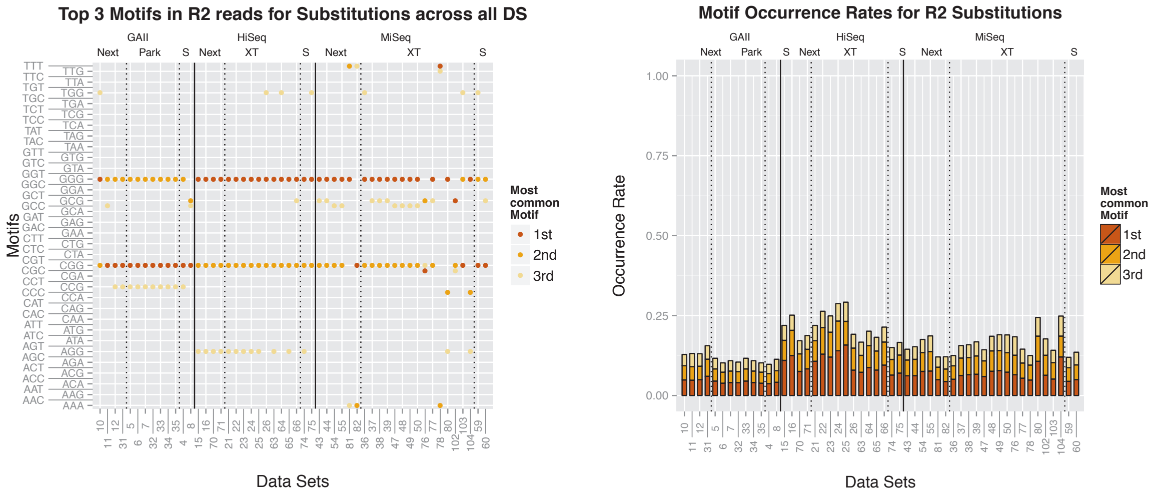

(a) R2 substitutions

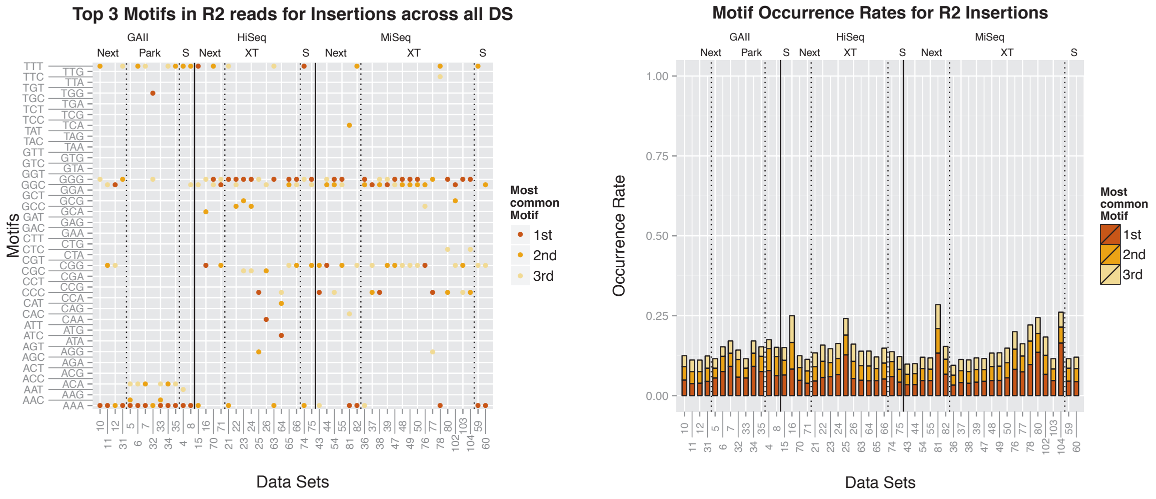

(b) R2 insertions

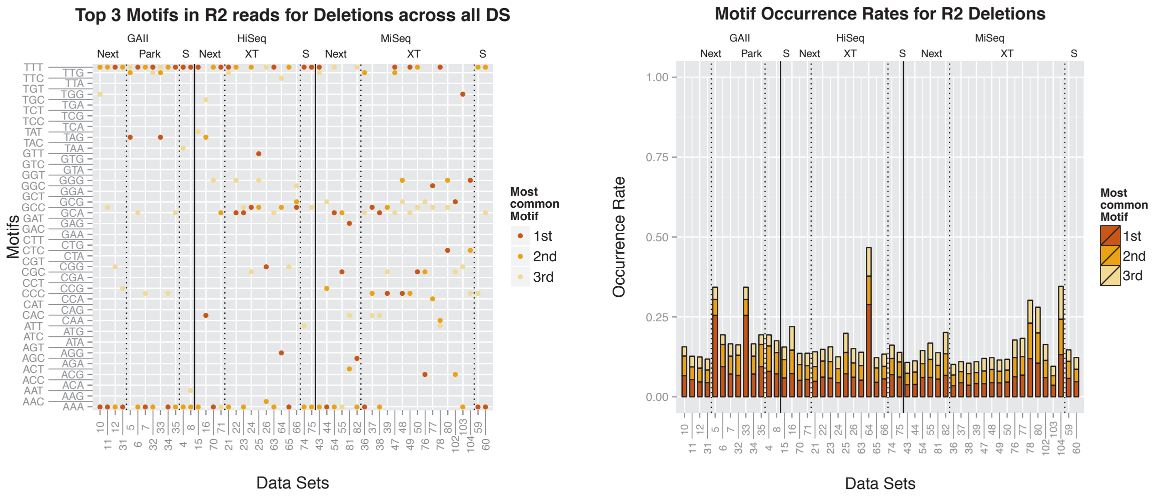

(c) R2 deletions

Figure S6 The top three motifs (3mers preceding errors) for R2 substitutions, insertions and deletions are displayed on the left. The rates associated with each motif are displayed on the right. Data sets are grouped by sequencing platform and library preparation method.

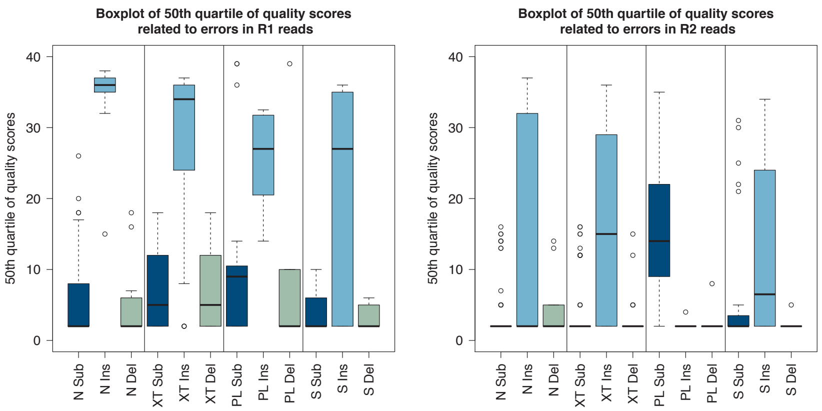

Figure S7 Overview of the 50th quartile of quality scores associated with errors across all data sets. The results for the R1 reads are displayed on the left and the results for the R2 reads are on the right. Data sets were grouped by library preparation method (N = Nextera, XT = NexteraXT, PL = Parkinson, S = Standard TruSeq) and substitution, insertion an deletion errors are displayed separately.

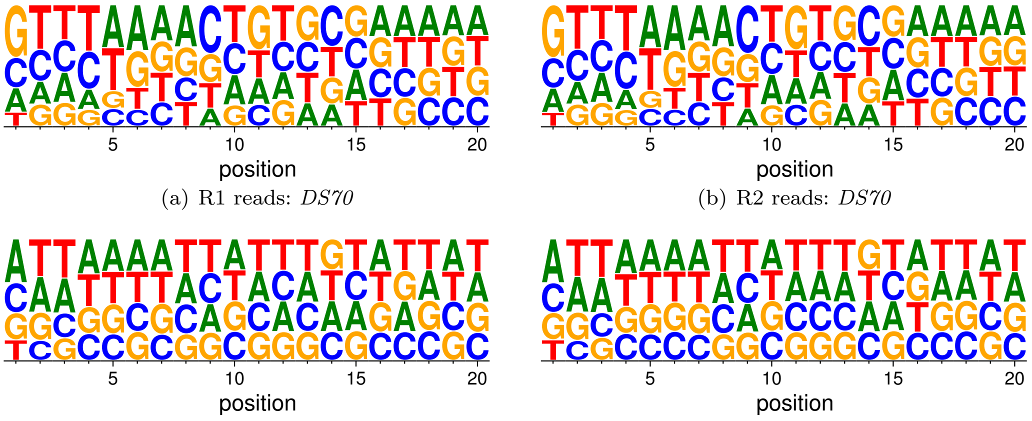

(a) R1 reads: *DS70*

(b) R2 reads: *DS70*

(c) R1 reads: *DS74*

(d) R2 reads: *DS74*

Figure S8 Transposome insertion bias: The figure displays the nucleotide representation across the first 20bp for the R1 and R2 reads of data set *DS70* and *DS74*, respectively. The library for *DS70* was prepared with the Nextera method and sequenced on a HiSeq; *DS74* was prepared with the standard TruSeq kit and sequenced on a HiSeq.

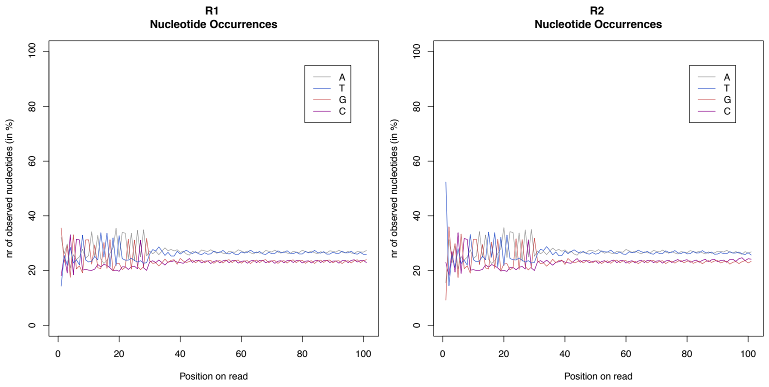

Figure S9 Nucleotide rates: Comparison of occurrence rates of the four nucleotides across the reads for data set *DS6*. The library for this data set was prepared with the Parkinson method and sequenced on the GAII. Fluctuations were observed at the read start affecting 30bp.

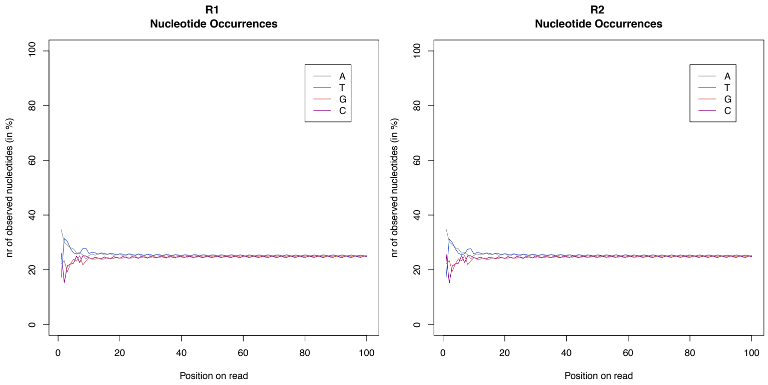

Figure S10 Nucleotide rates: Comparison of occurrence rates of the four nucleotides across the reads for data set *DS74*. The library for this data set was prepared with the standard TruSeq method and sequenced on the HiSeq. Fluctuations at the read start are much smaller compared to the transposome-based library technologies and affected a smaller number of bases.

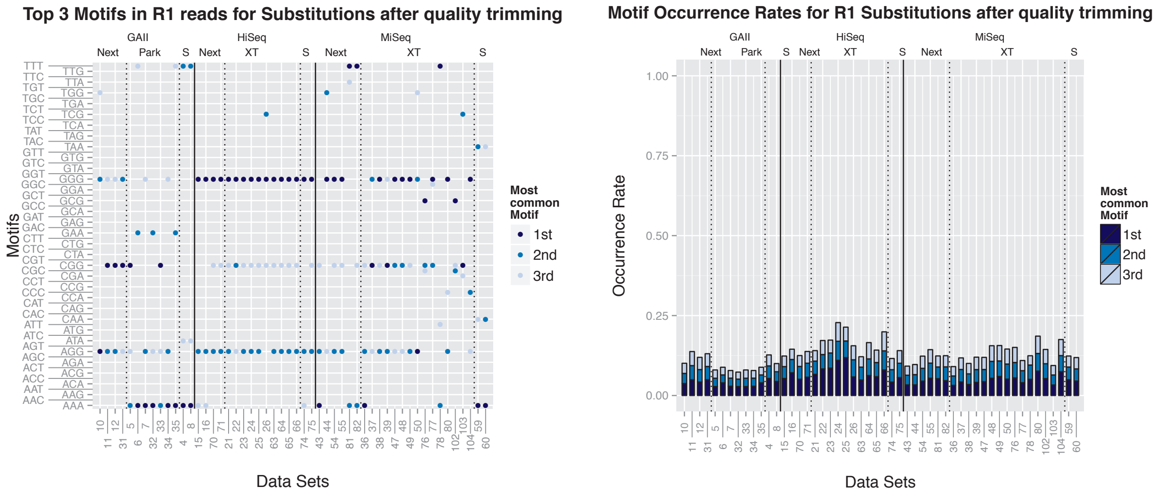

(a) R1 substitutions after quality trimming

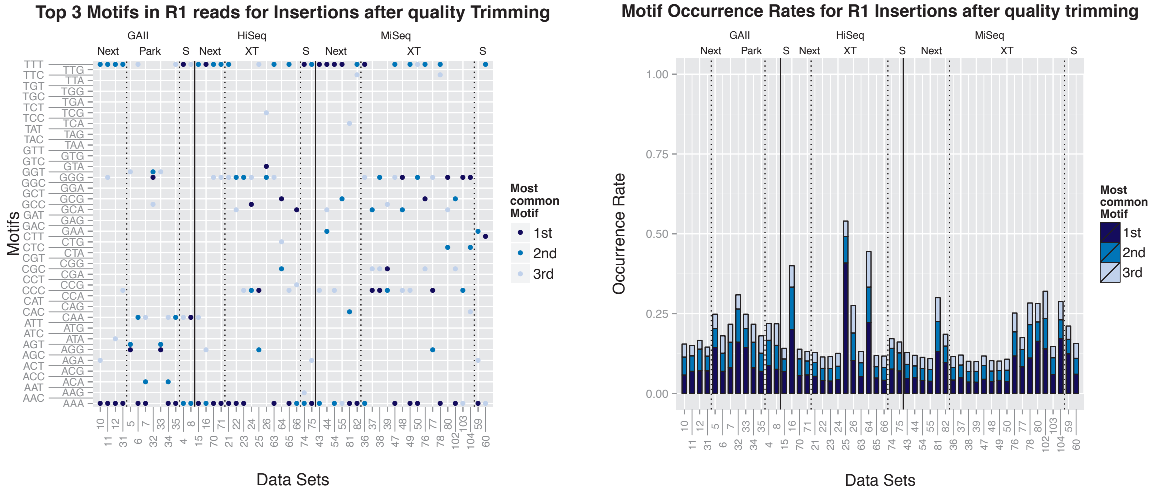

(b) R1 insertions after quality trimming

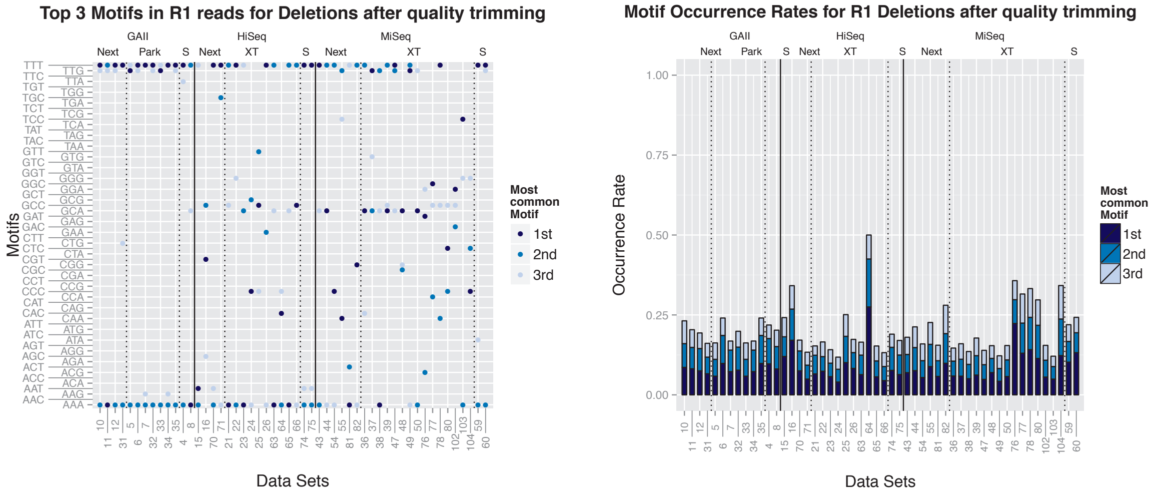

(c) R1 deletions after quality trimming

Figure S11 Motifs after quality trimming: The top three motifs (3mers preceding errors) for R1 substitutions, insertions and deletions are displayed on the left. The rates associated with each motif are displayed on the right. Data sets are grouped by sequencing platform and library preparation method.

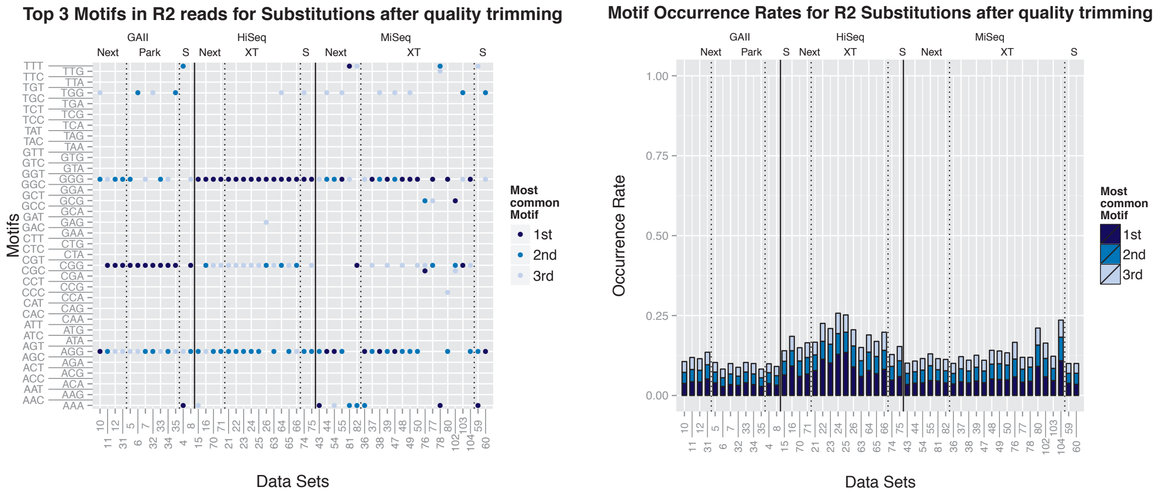

(a) R2 substitutions after quality trimming

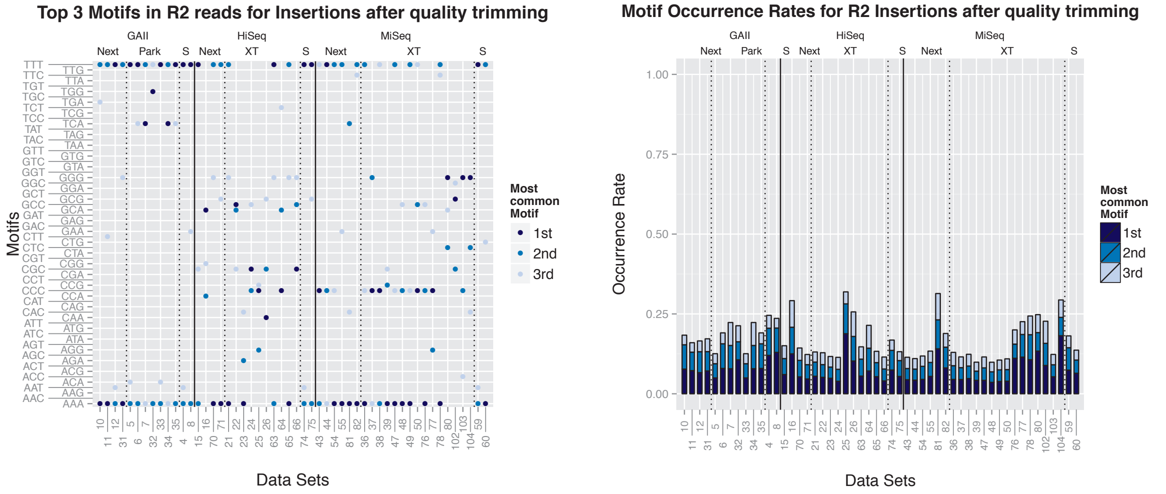

(b) R2 insertions after quality trimming

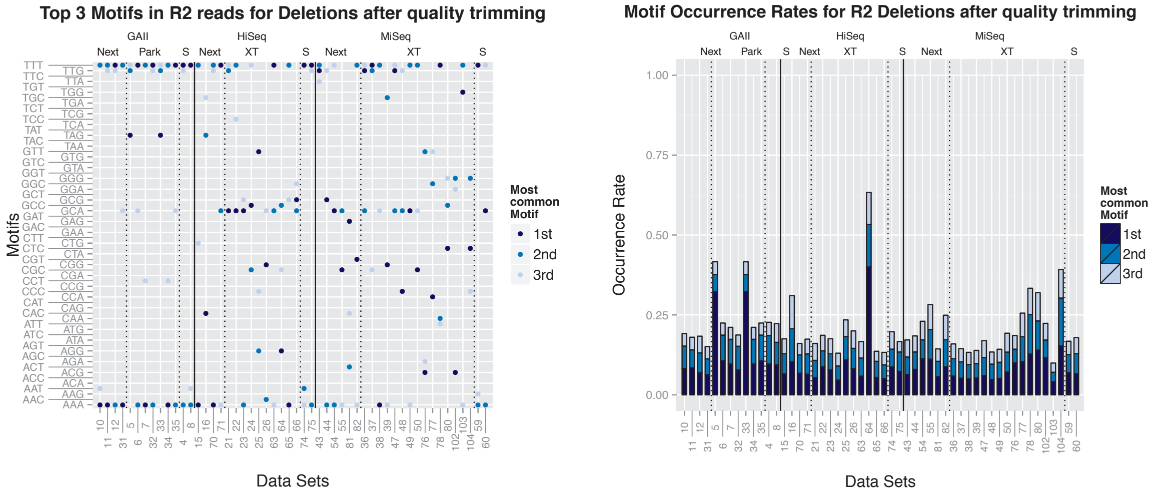

(c) R2 deletions after quality trimming

Figure S12 Motifs after quality trimming: The top three motifs (3mers preceding errors) for R2 substitutions, insertions and deletions are displayed on the left. The rates associated with each motif are displayed on the right. Data sets are grouped by sequencing platform and library preparation method.

Table S2 Overview of the most common motifs implicated in substitutions for GAII, HiSeq and MiSeq after quality trimming.

Platform	R1/R2	1st motif	2nd motif	3rd motif
**GAII**	R1	AAA/CGG	AGG	AGG/GGG
**GAII**	R2	CGG	AGG/GGG	AGG
**HiSeq**	R1	GGG	AGG	CGG
**HiSeq**	R2	GGG	AGG	CGG
**MiSeq**	R1	GGG	AGG	CGG
**MiSeq**	R2	GGG	AGG	CGG

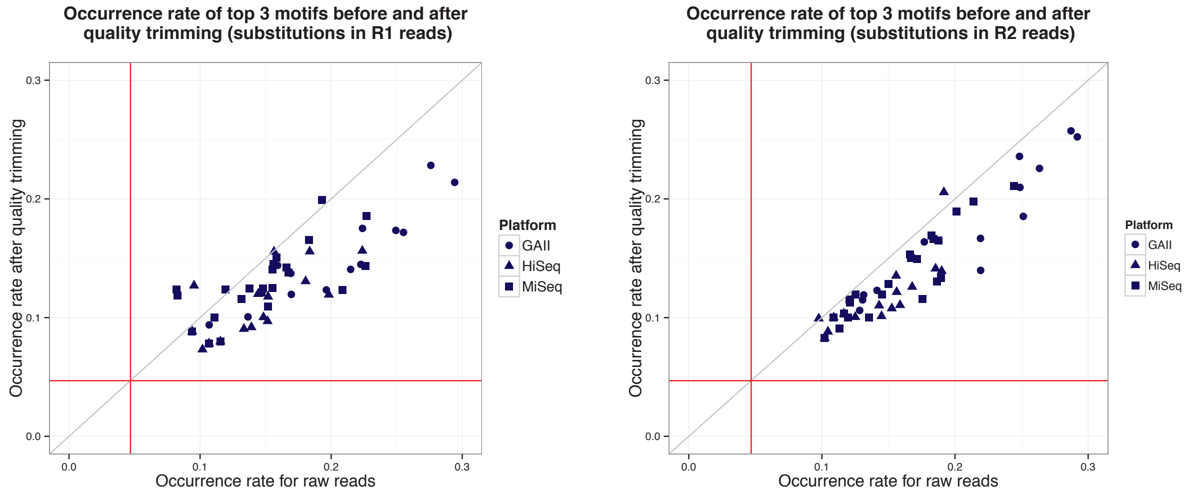

(a) R1 substitutions (b) R2 substitutions

Figure S13 Comparison of motif occurrence rates before and after quality trimming: The figure displays the occurrence rates of the top three motifs in connection with R1 and R2 substitutions of the raw reads (x-axis) and after quality trimming (y-axis). The red lines indicate the expected accumulative occurrence rate for three randomly occurring motifs. Although the relative number of substitutions connected to the top three motifs decreases after quality trimming for most samples, the strong motif bias remains.

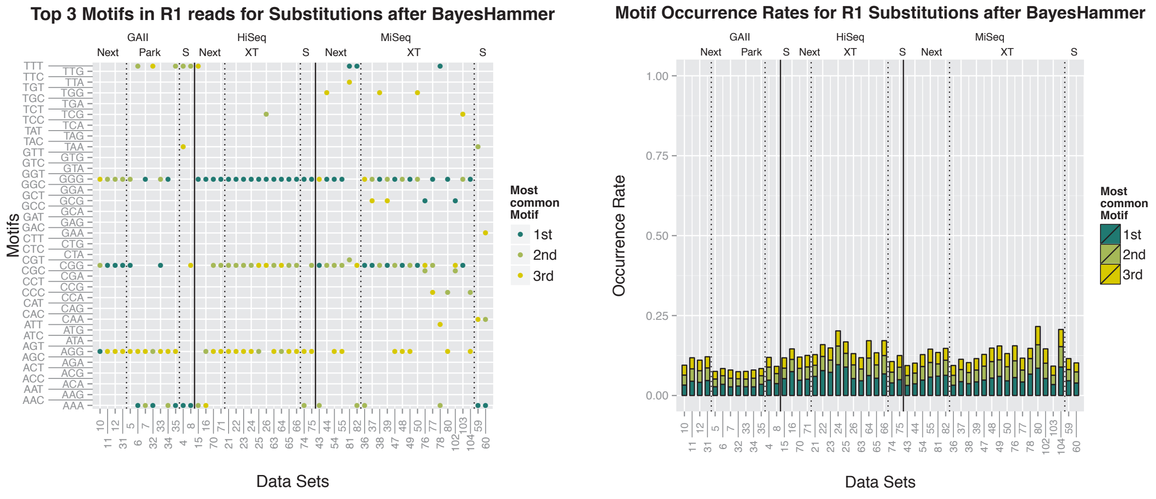

(a) R1 substitutions after BayesHammer

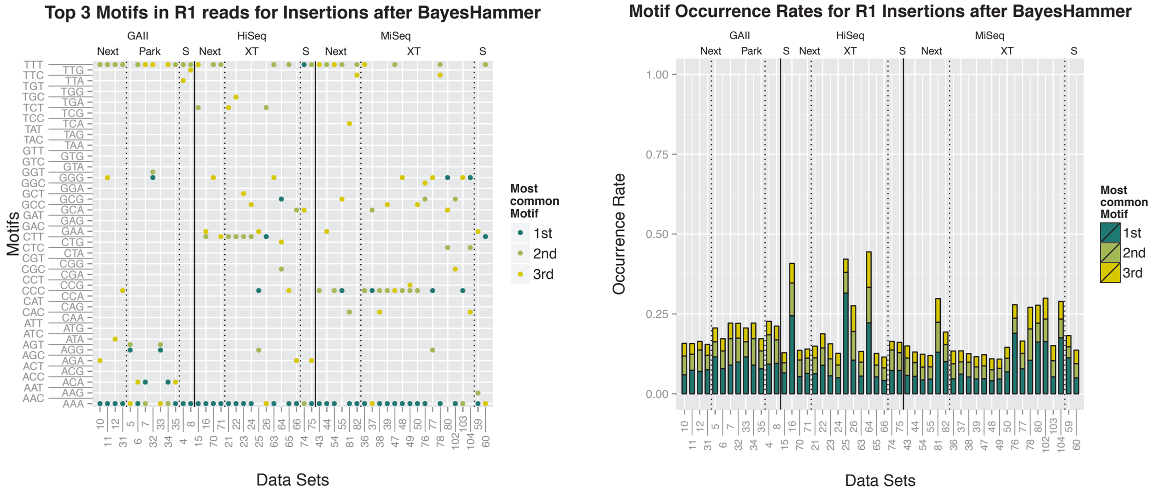

(b) R1 insertions after BayesHammer

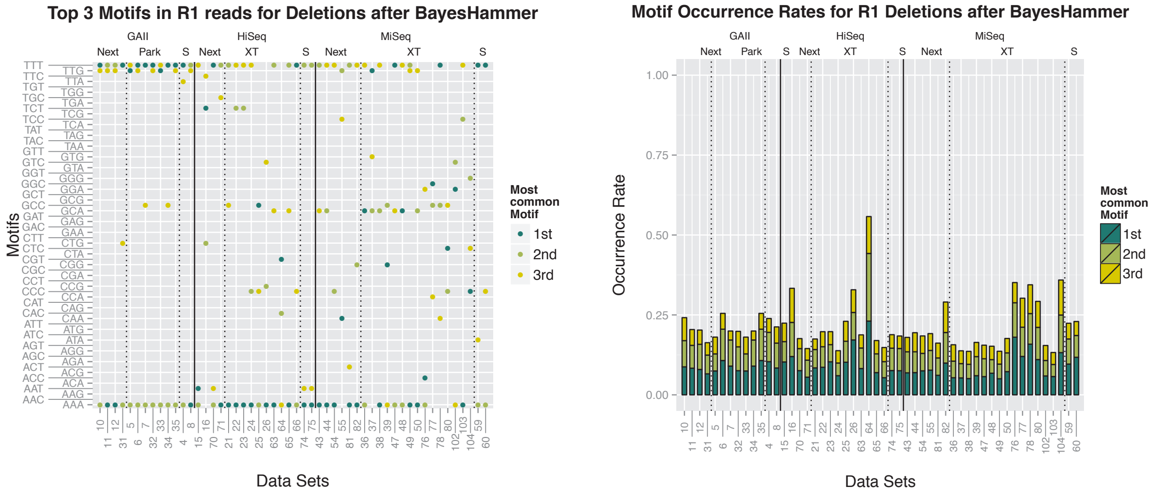

(c) R1 deletions after BayesHammer

Figure S14 Motifs after BayesHammer: The top three motifs (3mers preceding errors) for R1 substitutions, insertions and deletions are displayed on the left. The rates associated with each motif are displayed on the right. Data sets are grouped by sequencing platform and library preparation method.

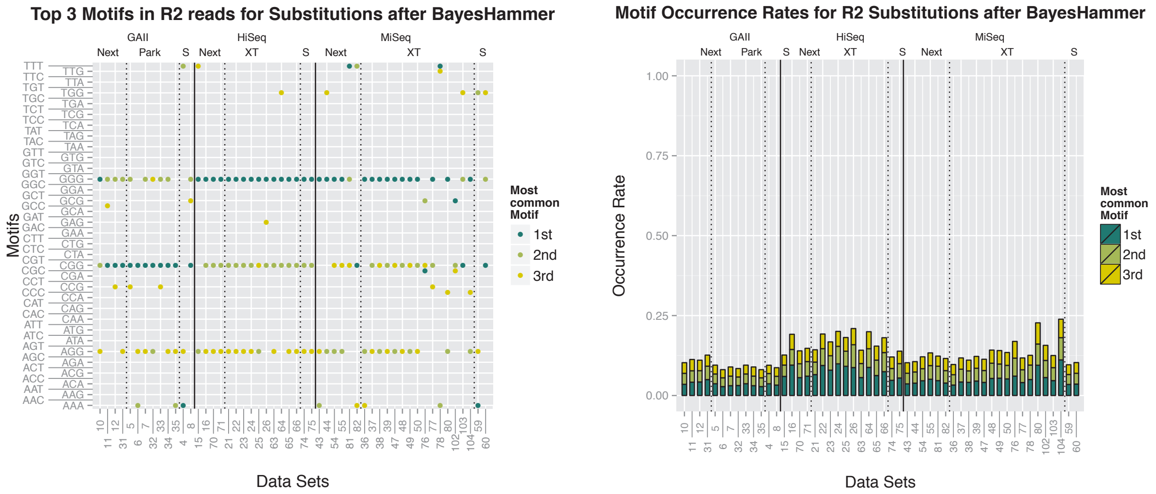

(a) R2 substitutions after BayesHammer

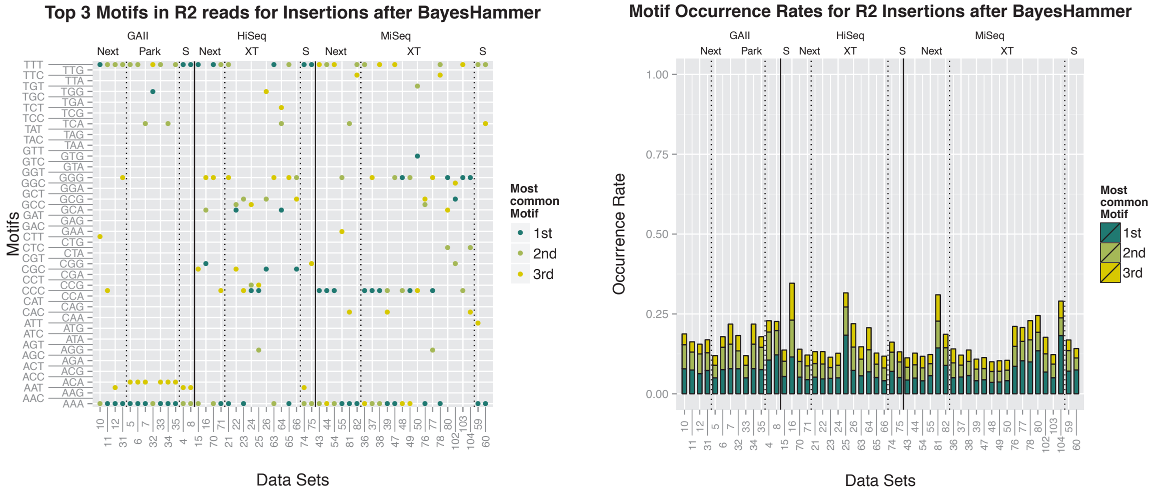

(b) R2 insertions after BayesHammer

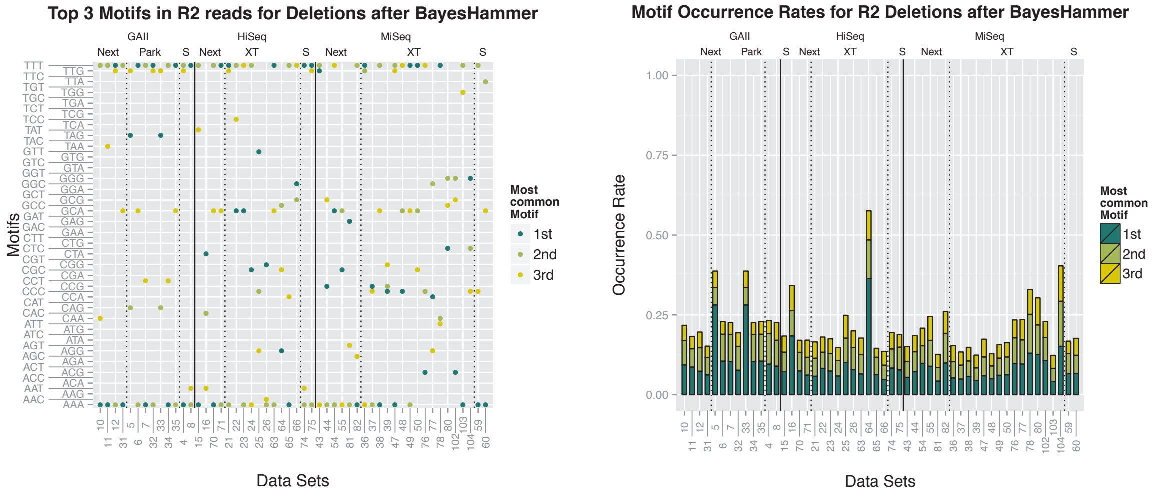

(c) R2 deletions after BayesHammer

Figure S15 Motifs after BayesHammer: The top three motifs (3mers preceding errors) for R2 substitutions, insertions and deletions are displayed on the left. The rates associated with each motif are displayed on the right. Data sets are grouped by sequencing platform and library preparation method.

Table S3 Overview of the most common motifs implicated in substitutions for GAII, HiSeq and MiSeq after error correction with BayesHammer.

Platform	R1/R2	1st motif	2nd motif	3rd motif
**GAII**	R1	AAA/CGG	GGG	AGG
**GAII**	R2	CGG	GGG	AGG
**HiSeq**	R1	GGG	AGG	CGG
**HiSeq**	R2	GGG	CGG	AGG
**MiSeq**	R1	GGG	CGG	AGG
**MiSeq**	R2	GGG	AGG	CGG

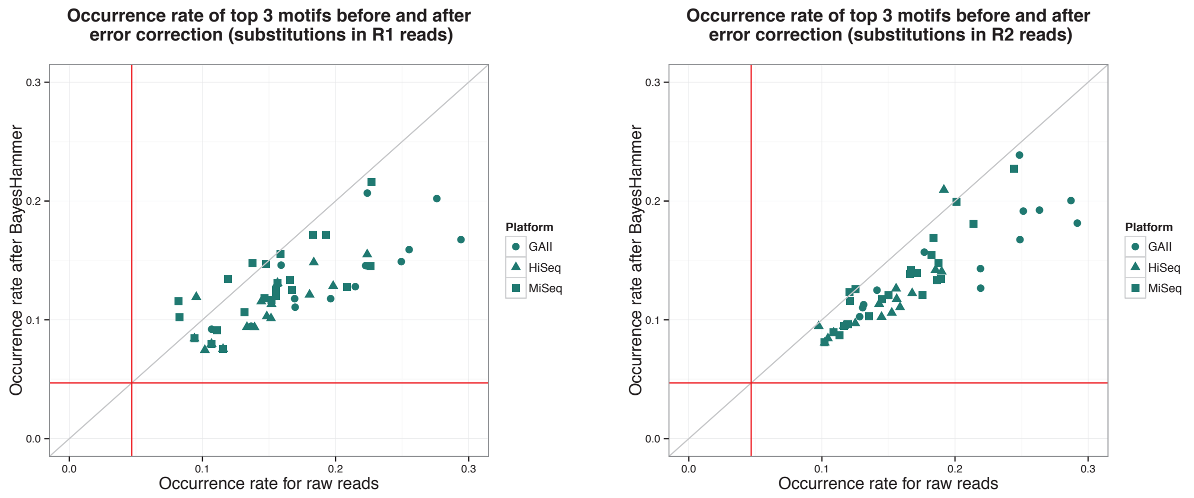

(a) R1 substitutions (b) R2 substitutions

Figure S16 Comparison of motif occurrence rates before and after error correction with BayesHammer: The figure displays the occurrence rates of the top three motifs in connection with R1 and R2 substitutions of the raw reads (x-axis) and after error correction (y-axis). The red lines indicate the expected accumulative occurrence rate for three randomly occurring motifs. Although the relative number of substitutions connected to the top three motifs decreases after error correction for most samples, the strong motif bias remains.

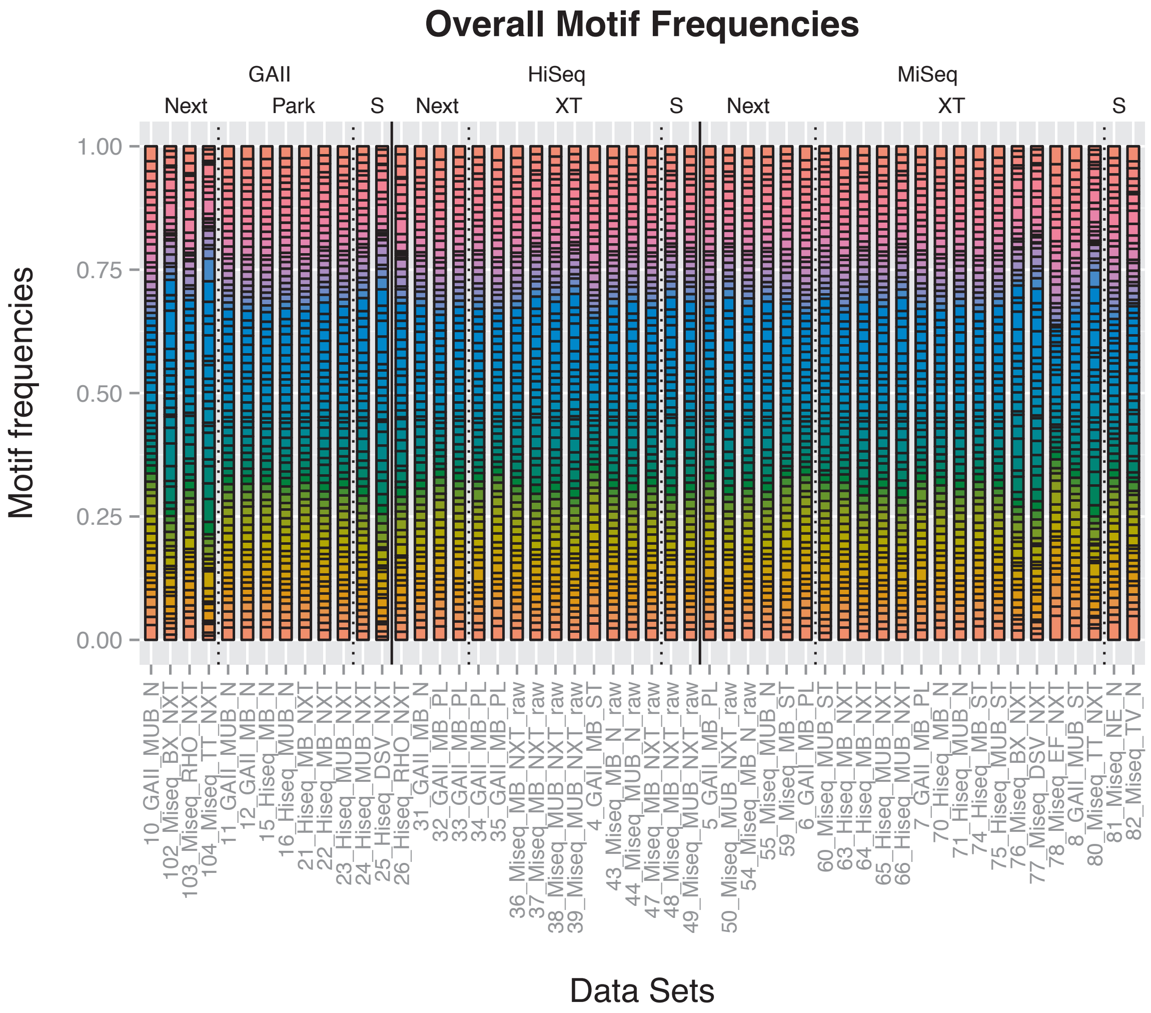

Figure S17 Motif frequencies in R1 reads: The figure displays the frequencies of all 64 3mers in the R1 reads for each of the data sets. Motifs are in the same order as displayed on the y-axis of plots in the left column of Figure S15. A non-uniform distribution of the 3mers is observed.

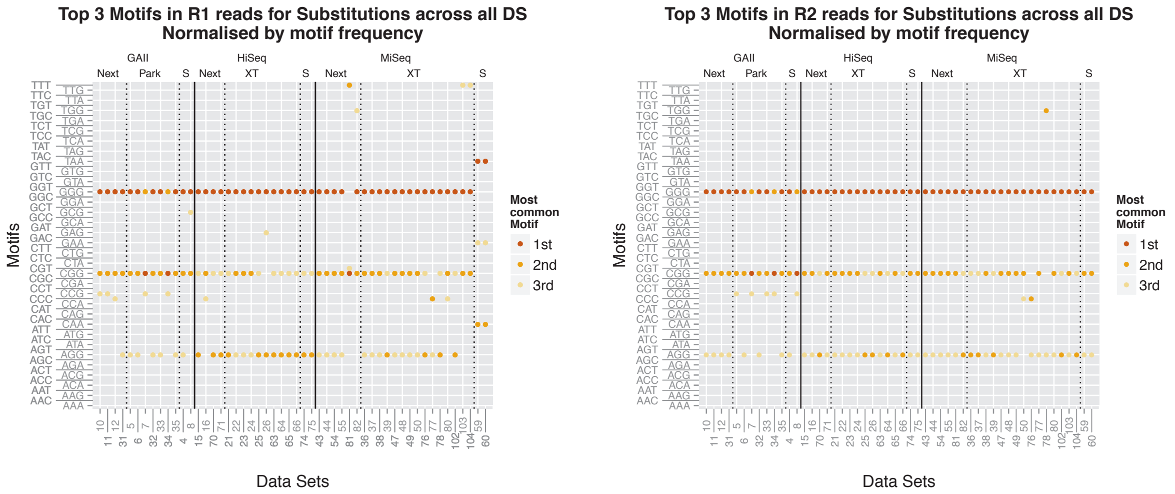

(a) Substitutions

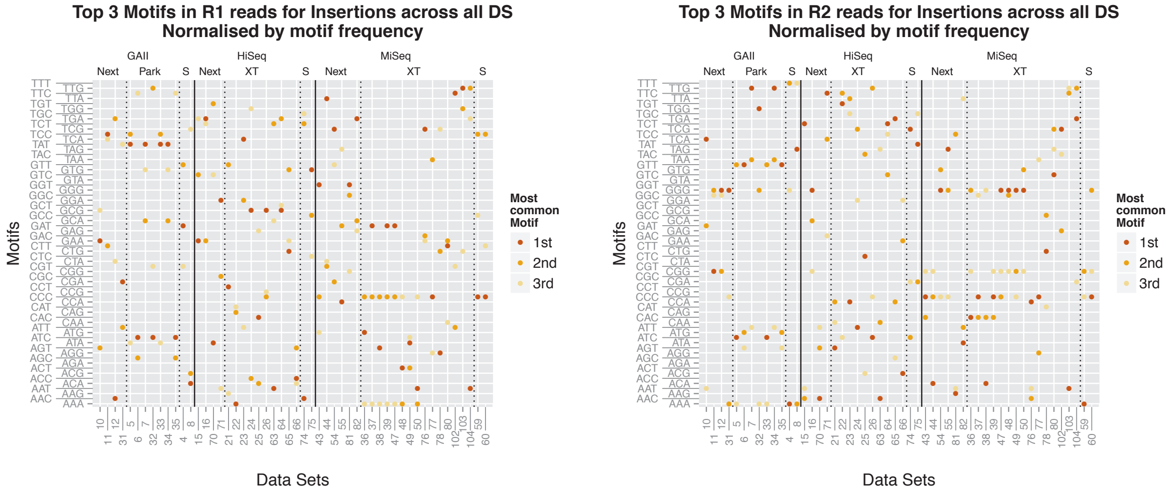

(b) Insertions

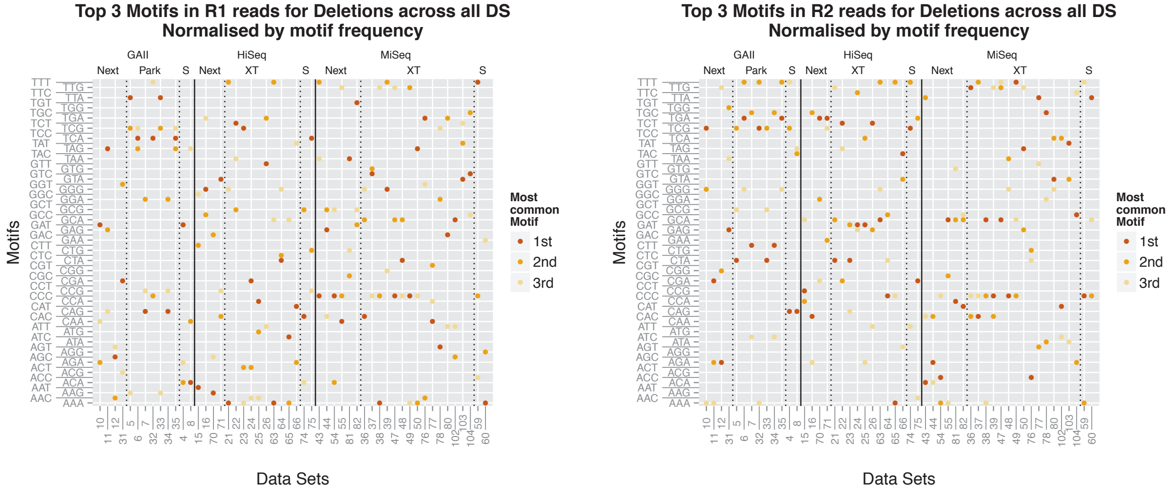

(c) Deletions

Figure S18 Comparison of motif occurrence rates normalized by motif frequencies: The figure displays the top three motifs in connection with R1 and R2 errors after normalization by motif frequency.
